# A co-culture model of the bovine alveolus

**DOI:** 10.12688/f1000research.18696.2

**Published:** 2019-07-30

**Authors:** Diane Lee, Mark Chambers

**Affiliations:** 1 School of Veterinary Medicine, University of Surrey, Guildford, Surrey, GU2 7AL, UK

**Keywords:** Replacement, NC3Rs, bovine, alveolus, type II, endothelial, air-liquid interface

## Abstract

The epithelial lining of the lung is often the first point of interaction between the host and inhaled pathogens, allergens and medications. Epithelial cells are therefore the main focus of studies which aim to shed light on host-pathogen interactions, to dissect the mechanisms of local host immunity and study toxicology. If these studies are not to be conducted exclusively *in vivo, *it is imperative that *in vitro *models are developed with a high *in vitro*- *in vivo* correlation. We describe here a co-culture model of the bovine alveolus, designed to overcome some of the limitations encountered with mono-culture and live animal models. Our system includes bovine pulmonary arterial endothelial cells (BPAECs) seeded onto a permeable membrane in 24 well Transwell format. The BPAECs are overlaid with immortalised bovine alveolar type II epithelial cells and cultured at air-liquid interface for 14 days before use; in our case to study host-mycobacterial interactions.

Characterisation of novel cell lines and the co-culture model have provided compelling evidence that immortalised bovine alveolar type II cells are an authentic substitute for primary alveolar type II cells and their co-culture with BPAECs provides a physiologically relevant *in vitro* model of the bovine alveolus.

The co-culture model may be used to study dynamic intracellular and extracellular host-pathogen interactions, using proteomics, genomics, live cell imaging, in-cell ELISA and confocal microscopy. The model presented in this article enables other researchers to establish an *in vitro* model of the bovine alveolus that is easy to set up, malleable and serves as a comparable alternative to *in vivo* models, whilst allowing study of early host-pathogen interactions, currently not feasible *in vivo*. The model therefore achieves one of the 3Rs objectives in that it replaces the use of animals in research of bovine respiratory diseases.

Research highlights ***Scientific benefits:***

- May reduce the requirement to conduct multiple *in vivo* studies.
- Immortalisation of cells allows consistency of data between studies.
- Easy set-up allows studies which cannot feasibly be performed in live animal models.
- Allows first-line investigations of specific disease pathways *in vitro*, hence is suited to drug discovery screens and toxicology studies.

***3Rs benefits:***

- Provides an alternative to *in vivo* experiments on bovine respiratory diseases, negating mild-moderate invasive procedures. Up to 100 cows per year (UK) are used in bovine respiratory studies, approximately 40 of which will be used in BTB vaccine studies (Annual Statistics of Scientific Procedures on Living Animals for Great Britain, 2017).

***Practical benefits:***

- Enables users to obtain a representative alveolar model with desirable physiological and structural features of the native bovine alveolus within 3 weeks.
- Immortalised cell lines facilitate reproducibility and comparisons with previously published literature.
- Cost effective compared to performing similar experiments *in vivo*.
- Enables researchers without animal facilities to study bovine respiratory diseases.

***Current applications:***

- The immortalised cell lines are suitable for studies involving monocultures of alveolar type II cells, whilst the co-culture may be used for transport studies of candidate pathogens, drug molecules, vaccines and interactions thereof.
- Currently used by researchers to study early host-pathogen interactions of *Mycobacterium bovis* and the live attenuated vaccine Bacillus Calmette–Guérin (BCG) in conjunction with peripheral blood mononuclear cells.

***Potential applications:***

- The co-culture model could potentially incorporate bovine macrophages and also be used to investigate the role of surfactant proteins in innate immunity.
- Sets the standard to recapitulate structural and functional features for future *in vitro* models of the alveolus.
- The co-culture alveolus model may be translatable to other species, with minor modifications.

## Introduction

The lung is a complex organ, lined in its entirety by specialised epithelium. The most distal region of the lung consists of the alveoli, designed primarily for efficient gas exchange and arranged in clusters or acini ^1^. The alveolar epithelium is composed of two types of cell; alveolar type I (ATI) and alveolar type II (ATII). Alveolar type I cells cover 90 % of the alveolar surface ^2^, despite only constituting in the region of 7 % of the epithelium by numbers ^3^. This can be attributed to the elongated squamous cell morphology of the ATI, which spreads over a large surface area and lies in close proximity to capillary endothelial cells. This lends the ATI to its primary role of gas exchange and enables the regulation of physiological solute transport between the alveolus and the circulatory system ^4^. Previous studies have provided evidence for the role for ATI cells in fluid homeostasis of the alveolar compartment, with the finding that sodium ions are transported via the epithelial sodium channel (ENaC), using active ion transport ^5^.

The ATI cell is terminally differentiated, being derived from the alveolar type II (ATII) cell. Unlike the former, the ATII cell is compact and ‘cuboidal’. Early studies performed in simians and rodents generated evidence to suggest that ATII cells expressing surfactant protein C are the main progenitor cells of the alveolar epithelium ^6– 8^. This role was cemented by the findings of later research, including lineage tracing studies ^9^ and morphological characterisation ^10^. Proliferation of the ATII cell is normally relatively slow compared to other epithelial cells ^9^; however ATII hyperplasia has been reported in response to injury, such as that inflicted by the chemotherapeutic agent bleomycin, providing further evidence that ATII cells are the main progenitor of the lung ^11^.

The close proximity of alveolar type I epithelial cells to the neighbouring capillary endothelial cells forms a highly gas permeable bilayer barrier across which gas exchange occurs. The structural integrity of the alveolus is maintained during its expansion and contraction by the formation and continuous secretion of a phospholipid-rich film (pulmonary surfactant) from ATII cells that spreads and covers the alveolar epithelial cell surface.

Perhaps most pertinent to the study of host-pathogen interactions is the role of ATII cells in innate immunity, protecting the lungs against respiratory infection. The ATII cell was first coined as the ‘defender of the alveolus’ by Mason and Williams in 1977 ^12^, following numerous studies into the phospholipid content of the surfactant secreted by primary ATII. As with mucus of the upper airways, the composition of surfactant varies according to disease status ^13, 14^. In particular, surfactant protein D (SP-D) has been hallmarked as a biomarker for pulmonary disease on account of its variability when quantified from bronchoalveolar lavage (BAL) or serum ^15^. Along with surfactant protein A (SP-A), SP-D has been found to bind to viruses, bacteria, yeast and fungi. Both act as opsonins, thus increasing the pathogen’s susceptibility to phagocytosis ^16– 19^.

It follows that further study of host-pathogen interactions of pathogens with ATII cells is critical to advances in the field. Costs, low throughput and ethical concerns are all limitations of *in vivo* models. In addition, early events at the host-pathogen interface cannot be studied easily *in vivo*, due to the transient nature of such early events. These limitations may be overcome by *in vitro* systems; however, few systems exist that accurately reproduce the structural complexity of the *in vivo* environment, particularly for non-human species. A human bilayer model of the alveolus was first described in 1999 ^20^ and was used to study early events in *Mycobacterium tuberculosis* infection. However, no equivalent *in vitro* model of the bovine alveolus is available to enable comparative studies between species, according to a search of the PubMed database using the terms, ‘bovine’, ‘alveolus’, ‘bilayer’, ‘co-culture’ and ‘model’ (latest search, June 2019). *In vivo* models of *M. bovis* and *M. tuberculosis* infection result in ‘non-recovery’ of subjects and thus raise strong ethical considerations ^21^. Cattle are natural hosts of *M. bovis* and are therefore one of the target species (alongside wildlife reservoirs such as badgers and wild boar) for strategies aimed at the study and control of bovine TB. It follows that the most relevant host-pathogen interaction studies would be performed by experimental infection of cattle. Furthermore, the close relationship of *M. bovis* to *M. tuberculosis* ^21^ gives rise to synergy in the efforts to find novel vaccines and therapeutic interventions to control human and bovine TB. For example, vaccines which were developed for use in humans have been evaluated in the bovine *M. bovis* live animal model with reported success, whilst immune responses and reaction to therapeutic strategies are also comparable ^22– 24^. The relatively large size of cattle, however, presents a major challenge. They require particularly specialist husbandry and housing at biosafety level 3 containment, which counter the advantages of frequent and sequential sample collection and infringe upon the ‘Five Freedoms’, as defined by the Farm Animal Welfare Council ^25^.

Continuous epithelial cell lines offer the advantage of reduced inter-experiment variability, extended proliferative capacity and better suitability for high-throughput screening. Such a cell line was generated from bovine ATII cells by Su *et al.* (2013) ^26^. However, upon enquiry, this was found to be no longer available. We have therefore developed a simple method of isolation for ATII cells ^27^ and generated two new cell lines, the bovine alveolar type II (BATII) and the bovine type 2 alveolar epithelial (B2AE) cell lines. These ATII derived cells have been integrated into an *in vitro* co-culture model, with bovine pulmonary arterial endothelial cells (BPAECs). Cultured at an air-liquid interface, this co-culture model recreates the fundamental elements of the bovine pulmonary alveolus.

Our model advances the replacement of cattle by providing a readily available and reproducible means to study fundamental events following infection of the bovine lung with virulent pathogens that cannot be conducted currently *in vitro*. The model is also amenable to translational and applied research, screening and toxicity studies, especially involving zoonotic pathogens, such as *M. bovis* and *M. tuberculosis,* or respiratory syncytial virus, the human and bovine strains of which are closely related. The ease of set up and small footprint per experiment also lends the model well to the generation of pharmacological data for novel therapies targeted at respiratory disease or topical agents delivered via inhalation. This applies to diseases such as bovine respiratory disease (BRD), for which new antimicrobial therapies are urgently needed in order to develop therapeutic strategies and perform pharmacokinetic studies ^28^. In the UK alone, the Annual Statistics of Scientific Procedures on Living Animals (2016) reports that a total of 95 procedures were performed on live cattle for respiratory research ^29^. We can, therefore, propose with confidence that upwards of this number could potentially be replaced, per year, globally.

## Methods

### Isolation of alveolar type II (ATII) epithelial cells

The isolation procedure is outlined in detail by Lee *et al.* (2018) ^30^. All efforts were made to ameliorate animal suffering. This was achieved in the current study by performing the isolation procedure on tissue sourced from freshly slaughtered cattle less than 24 months of age at a local abattoir facility, under existing licensed slaughter procedures. Details of the reagents required for isolation and culture of ATII cells may be found in Table 1. A 5 cm ^3^ piece of distal right lung was taken for disease surveillance. Tissue was dissected into pieces no greater than 3 mm ^3^ and washed in Dulbecco’s Phosphate Buffered Saline (DPBS) containing 100 U/mL each of penicillin and streptomycin, with five repetitions, with a further two washes in DPBS containing the same concentration of penicillin and streptomycin as well as 2 mM EDTA. Digestion was carried out for 30 minutes at 37°C in DPBS containing 100 U/mL each of penicillin and streptomycin, 0.008% (w/v) elastase (Fisher Scientific, UK), 0.2% (w/v) collagenase (Fisher Scientific), 0.005% (v/v) DNAse Type I (2000 KU/mL; Sigma, St.Louis, MI, USA) and 0.05% (w/v) trypsin (Gibco). Enzymatic activity was neutralised with an equal volume of Dulbecco’s modified Eagle Medium/Ham’s F12 (DMEM/F12) (Gibco) containing 25% FBS (heat-inactivated, Gibco) and 0.01% (v/v) DNAse I. The cell suspension was filtered sequentially through 100, 50 and 25 μm filters (Biodesign™, distributed through Fisher Scientific) and spun at 300 × *g* for 10 minutes at room temperature, before resuspending in 1:1 DMEM/F12/Small Airway Growth Medium (SAGM) (Lonza, UK), 5% FBS and 0.025% (v/v) DNAse I.

**Table 1.** List of reagents required for isolation of alveolar type II cells, generation of lentivirus and assembly of co-culture model.

Reagent	Supplier	Supplier #
Endothelial Growth Medium (EGM-2)	Lonza	CC-3162
Small Airway Growth Medium Bulletkit	Lonza	CC-3118
DPBS Ca2+/Mg2+ free	Gibco	12037539
Recovery Cell Freezing Medium	Invitrogen	12648010
0.25 % trypsin/0.05 % EDTA	Life technologies	25200056
Trypan Blue	Life technologies	15250061
Transwell-CLEAR™ 8μm, 6.5 mm (0.33cm ^2^)	Corning	3422
Dextran Blue 2000	Sigma Aldrich	D5751
Formaldehyde solution	Sigma Aldrich	47608
Goat serum	Sigma Aldrich	S26-M
Triton X-100	Sigma Aldrich	X100
Prolong® Gold Antifade reagent with DAPI	Thermo Fisher	P36941
Vectashield Hardset mounting medium with phalloidin (TRITC)	Vector Laboratories	H-1600
Prolong® Gold Antifade reagent with DAPI	Thermo Fisher	P36941
Vectashield Hardset mounting medium with phalloidin (TRITC)	Vector Laboratories	H-1600
ViraPower™ II Lentiviral Gateway® Expression Kit	Invitrogen	K367-20
Gateway Cloning Technology system	Invitrogen	12535-029
Opti-MEM I	Gibco	31985070
Lipofectamine 2000 Transfection Reagent	Invitrogen	31985062
Penicillin/Streptomycin 10 000 U/mL	Gibco	11548876
EDTA	Fisher Scientific	10030140
Porcine pancreas elastase	Fisher Scientific	15484279
Deoxyribonuclease I from bovine pancreas (DNAse Type I)	Sigma Scientific	D5025
Trypsin 0.25 %	Gibco	11560626
Collagenase Type I Clostridium histolyticum	Fisher Scientific	15434789
Matrix Filter Cell MicroSieve 100 mm pore	Fisher Scientific	12684787
Matrix Filter Cell MicroSieve 50 mm pore	Fisher Scientific	12924257
Matrix Filter Cell MicroSieve 25 mm pore	Fisher Scientific	12944257
IgG from bovine serum	Sigma Aldrich	I5506
Tris (hydroxymethylamine) methylamine	Fisher Scientific	10274300
Percoll	Sigma Aldrich	P1644
Foetal bovine serum, heat inactivated	Gibco	10270-106
Dulbecco’s modified Eagle Medium/Ham’s F12 (DMEM/F12)	Gibco	11320-074
SV40 Lentivirus particles	AMS Biotechnology	LVP557-GP
Ultimate Human Open Reading Frame (HORF) Clone TERT	Invitrogen	HORF01-IOH36343
Ultimate Human Open Reading Frame (HORF) Clone Bmi1	Invitrogen	HORF01-IOH13688
Bovine pulmonary arterial endothelial cells (BPAECs)	ECACC	86123101
Nunc Lab-Tek II 8 chamber coverglass slides	Thermo Fisher	Z734853

The cell suspension was overlaid onto petri dishes coated with bovine IgG (5 μg/mL; Sigma Aldrich), for 1 hour at 37°C, rocking after 30 minutes to redistribute non-adhered cells. Non-adherent cells were removed with the supernatant and spun at 300 × *g* for 5 minutes. The resulting pellet was resuspended in 4 % Percoll™ solution (Sigma Aldrich) and overlaid onto a gradient consisting of 30 % ‘heavy’ Percoll™ and 10 % ‘light’ Percoll™. Gradients were spun at 400 × *g* for 20 minutes at 4°C. Enriched ATII cells were identified as a band at the 10–30 % interface. These were removed and washed in DPBS containing 100 U/mL each of penicillin and streptomycin, resuspended in SAGM, plated onto 6-well plates and cultured at 37°C, 5 % CO _2_.

### Immortalisation procedure

Details of the reagents required for immortalisation of ATII cells may be found in Table 1. ATII cells were immortalised using lentivirus particles in two ways. The BATII cell line was generated by transduction with particles encoding the catalytic subunit of human telomerase (hTERT) and Simian Virus 40 large T antigen (SV40), whilst the B2AE utilised hTERT and the proto-oncogene B cell-specific Moloney murine leukemia virus integration site 1 (Bmi1). SV40 lentivirus was purchased as a ready to use particle suspension (AMS Biotechnology (Europe) Ltd, Abingdon, UK). To generate hTERT and Bmi1 lentiviral particles, the hTERT (human open reading frame clone pENTR221 IOH 36343) and Bmi1 (human open reading frame clone pENTR221 IOH13688) were purchased as Entry Vectors (Invitrogen Life Sciences, Carlsbad, CA, USA). The gene of interest was sub-cloned into a destination vector, in an LR Clonase™ recombination reaction using the Gateway Cloning Technology system (Invitrogen Life Sciences). This resulted in pLenti6-hTERT and pLenti6-Bmi1 expression vectors, or ‘expression clones’. The expression vectors were transformed into chemically competent One Shot Stbl3 *E. coli,* provided as a component of the Gateway Cloning Technology System. Insert integrity for both expression vectors was assessed by restriction enzyme digestion (XhoI) and sequencing. The latter was performed using primer sequences designed using the Primer-BLAST tool ^31^, generating primers directed at internal and flanking sites ( Table 2) in the expression vector.

**Table 2.** Primer sequences used to confirm expression vector integrity. Primers for the internal sequencing segments were designed using the Primer-BLAST online tool, whilst the external CMV and V5 (C-terminal) sequences were provided by Invitrogen.

Bmi1 Internal	FWD	ATCCCCACCTGATGTGTGTG
Bmi1 Internal	REV	TGTACAAGAAAGCTGGGTTC
hTERT Internal pair 1	FWD	CGACGTCTTCCTACGCTTCA
hTERT Internal pair 1	REV	CAAGAAAGCTGGGTTCTAGTCCA
hTERT Internal pair 2	FWD	GGAACCATAGCGTCAGGGAG
hTERT Internal pair 2	REV	GCTTCCCCAGGGAGATGAAC
CMV	FWD	CGCAAATGGGCGGTAGGCGTG
V5 (C-term)	REV	ACCGAGGAGAGGGTTAGGGAT

Live lentivirus particles were generated according to the protocols described in the Invitrogen Life Sciences ViraPower™ II Lentiviral Gateway® Expression Kit (Invitrogen Life Sciences). Briefly, 3 μg of pLenti6-hTERT or pLenti6-Bmi1 expression vector was mixed with 9 μg Virapower™ packaging mix in 1.5 mL Opti-MEM I medium (Invitrogen Life Sciences). This was added to 36 μL Lipofectamine™ 2000 transfection reagent (Invitrogen Life Sciences) (pre-diluted in 1.5 mL Opti-MEM I). The DNA-Lipofectamine complexes were added to a 10 cm tissue culture treated plate containing 5 mL of Opti-MEM I/10 % FBS. To this, 6 × 10 ^6^ 293FT producer cells (supplied with the kit) were added to 5 mL Opti-MEM I/10 % FBS, following Invitrogen’s reverse transfection protocol. Lentiviral particles were harvested as supernatant at 24, 48 and 72 hours post transfection and combined, before centrifugation at 300 × *g* for 15 minutes to pellet cell debris. The supernatant was then filtered through a 0.45 μm PVDF filter and centrifuged at 20,000 rpm, using a TH-641 rotor in a Sorvall WX80+ ultracentrifuge for 90 minutes at 4°C, to pellet viral particles.

Viral titre was determined using the HeLa cell line (p2 after receipt) according to the Virapower™ protocols and recommendations, using dilutions at 10 ^-2^, 10 ^-3^, 10 ^-4^, 10 ^-5^ and 10 ^-6^. HeLa cells were seeded at 2 × 10 ^5^ cells/well in a 6-well plate, in DMEM/10 % FBS/1 × non-essential amino acids (‘complete medium’, all Gibco). On the day of transduction (24 hours after seeding), cell supernatant was removed and the viral supernatant dilutions, prepared in complete medium, were added directly to the cell cultures (1 mL total culture volume). Medium only was added to a ‘mock’ well, used to confirm cell death in the presence of the selection antibiotic, blasticidin. To each well, 6 μg of Polybrene® transfection reagent (Sigma Aldrich) was added. Cells were returned to the incubator and cultured at 37°C, 5 % CO _2 _for 24 hours. Medium containing the viral supernatant was then replaced with 2 mL complete culture medium and the plates returned to the incubator for a further 24 hours. At 48 hours after transduction, blasticidin was added to all wells except untreated controls at a concentration of 10 μg/mL. Medium containing blasticidin was replaced every 3–4 days, until 14 days, when all cells were dead in the mock well and discrete antibiotic-resistant colonies could be seen in wells transduced with viral supernatant. These were counted following visualisation with 1 % crystal violet solution (Sigma Aldrich) and titre determined by averaging the counts of two wells, following correction for dilutions. Titred virus was stored at -80°C until use.

Primary ATII cells were immortalised at p1, five days after isolation. Cells were seeded into four wells of a 24-well plate at a density of 0.5 × 10 ^5^ cells/mL, 0.5 mL per well, in complete SAGM, without antibiotics. At 24 hours, SV40 and hTERT lentiviral particles were added simultaneously at a multiplicity of infection (MOI) of 10 (for each virus), in 0.5 mL SAGM, again without antibiotics. At 24 hours post infection, the media was replaced with fresh SAGM (1 mL). Cultures were fed every 2–3 days with SAGM and passaged accordingly, alongside primary ATII cells for comparison and grown without selection, monitoring morphology, proliferation rate and karyotype.

### Growth curves

Growth curves for the immortalised cell lines were performed at passages 2, 10 and 18, comparing them with those of primary ATII cells at passage 2, using the CellTitre 96® AQ _ueous_ One Proliferation Assay (Promega, Madison, WI, USA). Cell number and viability were determined by trypan blue exclusion (cells displaying uptake of trypan blue were considered non-viable) using a TC-20 automated cell counter (BioRad, Hercules, CA, USA). For each cell type/passage, cells were resuspended to a final concentration of 0.5 × 10 ^5^ cells/mL in SAGM. An aliquot (100 μL, 5000 cells) was dispensed into three wells of a 96-well plate (x 9 plates), before incubation in a humidified, 5 % CO _2_ atmosphere at 37°C. At 24 hours, one plate was removed. To each well, 20 μL of CellTiter 96® AQueous One Solution Reagent was added, returning the plate to the incubator for 1 hour. Absorbance was then measured at 490 nm, using the CLARIOstar® 96-well plate reader (BMG Labtech, Ortenburg, Germany). The assay procedure was repeated for the remaining time points. Resulting absorbances were entered as interpolated values against a reference curve generated from seeding pre-determined cell numbers in the same format and performing the assay after a 1hour incubation period.

### Karyotyping

To determine genetic integrity and characterise the stability of the immortalised cell lines, a metaphase chromosomal spread was carried out to ascertain karyotype of the BATII cell line at passages 4, 14 and 23 and of the B2AE cell line at passages 7, 12 and 22. The preparation of each spread was based on the protocol published by Campos *et al.* ^32^, with some deviations. Briefly, BATII, B2AE or wild-type ATII cells at the relevant passage were revived from liquid nitrogen stores and cultured for 48 hours in SAGM, in a humidified, 5 % CO _2_ atmosphere at 37°C. Cells were then treated with Colcemid™ (Sigma Aldrich) at a final concentration of 0.2 μg/mL in SAGM. Cells were returned to the incubator for 4 h, before trypsinisation with 0.25 % trypsin/0.05 % EDTA (Gibco/Invitrogen Life Sciences) for 5 minutes at 37°C. After trypsin neutralisation, cells were centrifuged at 122 × *g* for 5 minutes. The supernatant was discarded and 5 mL pre-warmed (37°C) hypotonic solution (KCl 75 mM _(aq)_) was added in a two-stage process: 3 mL was added to the tube in an inclined position, whilst rotating the tube to mix cells, followed by the remaining 2 mL to the upright tube. The cells and hypotonic solution were placed at 37°C for 15 minutes to allow swelling. To fix cells, three drops of room temperature fixative solution (freshly prepared methanol/glacial acetic acid 3:1) were added to the tube, followed by inversion to mix and centrifugation (without brake) at 122 × *g* for 5 minutes. The supernatant was again discarded, leaving 200 μL hypotonic solution. Fixative solution (3 mL) was added to the tube at an incline, rotating as before, followed by a further 2 mL to the tube wall, to wash attached cells down. The tube was stored upright overnight at 4°C, before centrifugation as before. Supernatant was again discarded, leaving 200 μL, and the pellet was resuspended by flicking the tube. Fixative solution (5 mL) was added as before and the centrifugation step repeated, again leaving 200 μL supernatant and resuspending by flicking the tube. An aliquot of each sample (20 μL) was spread onto a microscope slide pre-cleaned in 6M HCl, moving the pipette tip across the surface during dispensing. The slide was then passed through steam (face up). Slides were mounted with coverslips in Vectorshield Hardset with DAPI (Vector Laboratories, Burlingame, CA, USA) and examined under a fluorescence microscope to determine karyotype.

### Coating of plastic and Transwell inserts with Matrigel

To test the effects of an extracellular matrix on the culture of ATII cells, we coated tissue culture in plastic and also permeable inserts in a 10 % solution of growth factor reduced Matrigel™ (356230, Corning). Matrigel™ was thawed on ice overnight in a cold room and kept on ice throughout the procedure. Pre-cooled SAGM was stored on ice and Matrigel™ added to a final concentration of 10 %. Using chilled pipette tips, the solution was used to coat either 12 mm diameter (1.12 cm ^2^) Transwell 3.0 μm pore size PET inserts (3462, Corning, New York, US) or the surface of wells in a 24-well tissue culture plate (3337, Corning) (50 μL per cm ^2^). It was important to characterise the model using a large pore size, as we intend to use the co-culture model in future studies to study the migration of mycobacterial cells through the layers, analogous to studies in the nearest human equivalent model ^33^. Coating was performed overnight at 2–8°C and stored until use (up to 2 weeks). Coated surfaces were gently washed once with SAGM to remove excess Matrigel™.

### Culture conditions of BPAEC

Bovine pulmonary artery endothelial cells (BPAECs) were purchased from the European Collection of Authenticated Cell Cultures (ECACC) (Salisbury, UK) and cultured in endothelial growth medium (EGM-2 Bulletkit) (Lonza, UK). Cells were used between passages 3 and 10 only.

### Assembly of co-culture

**Figure 1.**
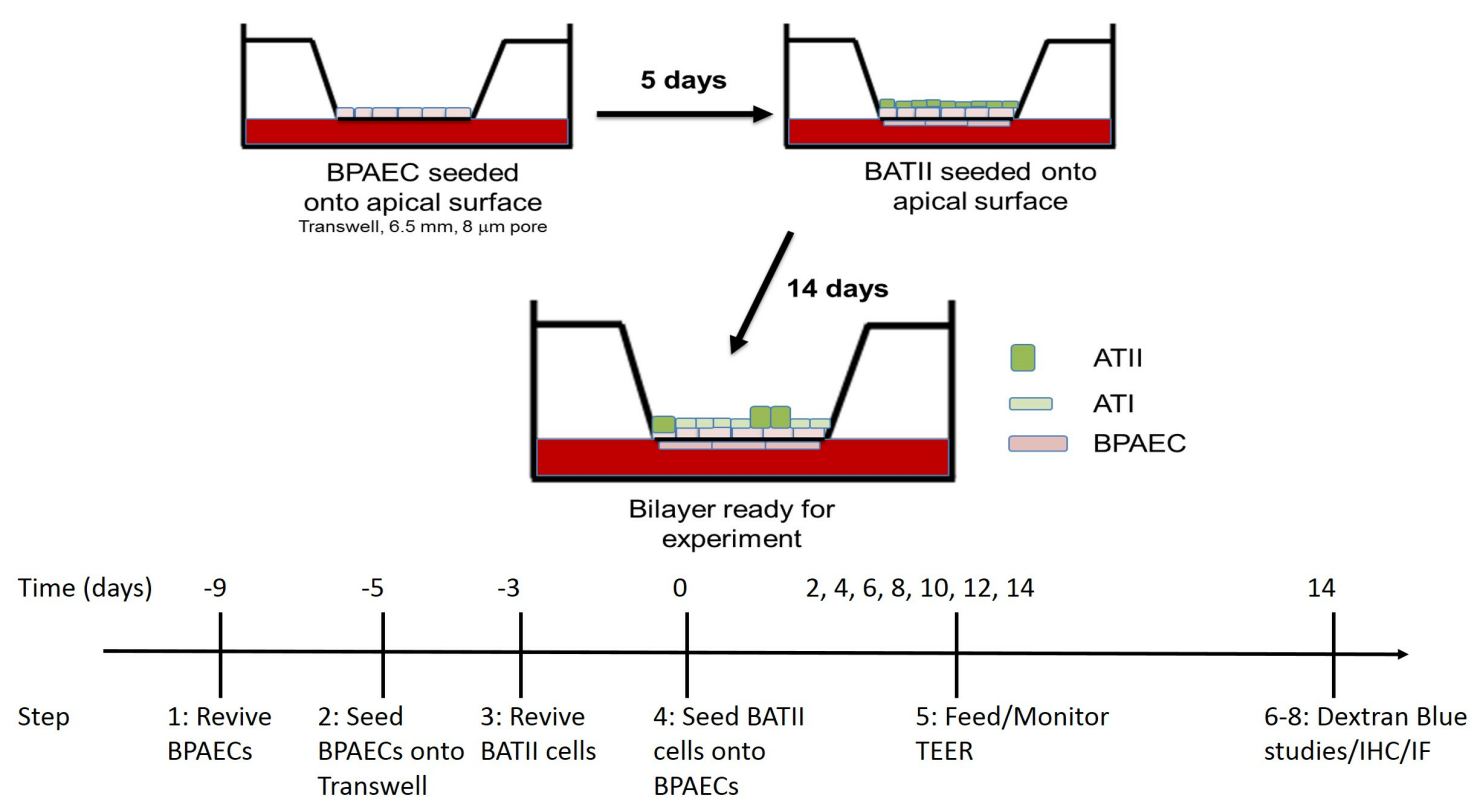
Schematic of the procedure used to set up a 14-day co-culture model. Bovine pulmonary arterial endothelial cells (BPAECs) are seeded onto the apical surface of the insert membrane and cultured for 5 days. The immortalised Bovine Alveolar Type II (BATII) epithelial cells are then overlaid onto the BPAECs and the co-culture cultured for 14 days as outlined in the methods section. Also shown is a timeline detailing the step(s) required at each timepoint.

A schematic of the co-culture assembly is shown in Figure 1. BPAECs were revived from liquid nitrogen and cultured to 80 % confluence in EGM-2. Cells were trypsinised using 0.25 % trypsin/0.05 % EDTA and neutralised using an equivalent volume of DMEM/10 % FBS, before seeding onto the apical surface of 6.5 mm diameter (0.33 cm ^2^) Transwell-CLEAR™ 3 μm pore size permeable membranes in a 24-well plate, at a density of 2 × 10 ^4^ cells/insert, (approximately 6.5 × 10 ^4^ cells/cm ^2^). EGM-2 (600 μL) was added to the basolateral chamber of each well. BPAECs were cultured for 5–7 days, replacing EGM-2 in the basolateral chamber and removing apical medium which had seeped through from the basolateral side of the membrane. BATII (or B2AE) cells were revived 3 days after BPAEC and cultured in SAGM without antibiotics. On the day of seeding, cells were trypsinised, resuspended in EGM-2 and counted, before seeding on top of the BPAEC layer at a density of 2 × 10 ^4^ cells/insert, as before. For histology, additional co-cultures were seeded onto 12 mm diameter (1.12 cm ^2^) Transwell-CLEAR™ 0.4 μm pore size (also 6.5 × 10 ^4^ cells/cm ^2^ for each cell line), adding 1.5 mL EGM-2 basolaterally. In each case, the co-culture was returned to the incubator for 2 hours to allow for attachment, after which the apical medium was removed and the cells cultured at air-liquid interface for 14 days, feeding every 2–3 days basolaterally (600 μL) and removing any medium on the apical surface. Monolayers were also prepared for each cell type, using the same seeding densities and culture methods/feeding intervals for comparison.

### Measurement of cell layer integrity

The formation of tight junctions was assessed for monolayers of both cell types and co-cultures in three ways. Firstly, each mono (BPAEC or BATII only) or co-culture was visually assessed, observing the apical surface by eye to estimate coverage by EGM-2 which had come through from the basolateral chamber.

Secondly, trans-epithelial electrical resistance (TEER) was measured between the time of seeding until the day of harvest. TEER values were measured using an EVOM2 Voltohmmeter with STX-2 chopstick electrodes (World Precision Instruments, Stevenage, UK) immediately before the medium was exchanged. For measurements, 0.5 mL and 1.0 mL of medium were added to the apical and basolateral chambers, respectively, allowing the medium to equilibrate to 37°C before measurements were performed in triplicate. All values were converted to Ohms/cm ^2^ using Equation 1.

$Final\;TEER\;(\Omega /cm^{\text{2}})=Net\;TEER\;(\Omega )\times \;Area\;of\;Transwell\;insert\;(cm^{\text{2}})\;(\text{Equation 1})$

The third method of determining layer integrity utilised the dextran permeability method, as outlined by Birkness *et al*. (1999) ^20^. A 10 mg/mL solution of Dextran Blue 2000 (DB2000; Sigma Aldrich) was prepared in DPBS, alongside standard solutions of 7.5, 5.0, 3.75, 2.5, 1.25, 0.625, 0.3125 and 0.156 mg/mL. DPBS (600 μL) was added to each well of a fresh 24-well plate. Inserts containing cells were removed one by one from the original plate, removing any apical medium, and transferred to the fresh plate. DB2000 (100 μL of 10 mg/mL) was added to the apical surface of each insert during the transfer. The plate was then placed at 37°C, 5 % CO _2_, for 1 hour. A blank insert was treated in the same way. An empty well had 600 μL DPBS added, followed by 100 μL of 10 mg/mL DB2000 in DPBS (total volume 700 μL). This was designated ‘no membrane control’. At the end of the incubation, 600 μL was removed from the basolateral compartment and read at 630 nm on a spectrophotometer, alongside the prepared standards. An aliquot (60 μL) was taken from apical solutions and diluted in 540 μL DPBS (a ten-fold dilution), before reading at 630 nm. Unknown values were extrapolated from a linear regression of the standard curve.

### Microscopy

For characterisation of the novel cell lines, BATII and B2AE cells were seeded onto Nunc Lab-Tek II 8 chamber coverglass slides (Thermo Fisher). Cells were seeded at 4 × 10 ^4^ cells per chamber and cultured for 72 hours. Slides were then washed with DPBS and fixed in 4 % PFA at room temperature for 15 minutes, before permeabilisation by washing in an immunofluorescence wash buffer (IF buffer) ( Table 3) containing 0.1 % triton X-100 for 15 minutes. Blocking was performed for 1 hour at room temperature in DPBS/ 5 % normal goat serum/ 0.1 % triton X-100. Primary antibodies were diluted in blocking buffer, applied to cells and incubated overnight at 4°C. Cells were rinsed three times with IF buffer and secondary antibody applied for 1 hour at room temperature in the dark. Cells were again rinsed three times with IF buffer and stored at 4°C until imaging, using a Nikon Eclipse Ti confocal microscope.

**Table 3.** Recipe for 10X Immunofluorescence (IF) buffer, used to wash and permeabilise cells. The following ingredients are added to ~475 mL reverse osmosis water and the resulting solution buffered to pH 7.4. Top up to 500 mL, then store at 2–8°C. Remove to room temperature before use and dilute 1:10 with reverse osmosis water.

Product	Product Number	Supplier	QTY
Sodium chloride (NaCl)	S9888	Sigma Aldrich, UK	38 g
Dibasic heptahydrate (Na _2_HPO _4_)	S9390	Sigma Aldrich, UK	9.38 g
Monobasic monohydrate (NaH _2_PO _4_)	S9368	Sigma Aldrich, UK	2.07 g
Sodium azide (NaN _3_)	S8032	Sigma Aldrich, UK	2.5 g
Bovine serum albumin (BSA)	A2153	Sigma Aldrich, UK	5 g
Triton X-100	X100	Sigma Aldrich, UK	10 mL
Tween 20	P1379	Sigma Aldrich, UK	2.05 mL

For immunofluorescence (IF) analysis of co-culture cultures, inserts were washed with DPBS and processed as for coverglass monolayer cultures. Following the final wash to remove the secondary antibody, the membrane was removed from the insert using a scalpel blade, then mounted in Prolong® Gold Antifade reagent with 4′,6-diamidino-2-phenylindole (DAPI) (Fisher Scientific) or Vectashield Hardset mounting medium with phalloidin (TRITC) (Vector Laboratories, Burlingame, CA, US) between two cover slips. Co-cultures were imaged using a Nikon Eclipse Ti confocal microscope.

Primary antibodies are detailed below in Table 4. Choice of markers was based on successful reports of their use to identify the ATII epithelial and endothelial cell types. Mouse and rabbit IgG1 isotype controls were used accordingly.

**Table 4.** List of antibodies used for immunofluorescence studies.

Antibody	Product Number	Supplier	RRID	Host	Clonality	Dilution
Pro-surfactant Protein C (proSP-C)	ab3786	Millipore	AB_91588	Rabbit	polyclonal	1:100
Cluster of differentiation 74 (CD74)	sc47742	Santa Cruz	AB_627172	Mouse	Pin.1	1:100
Solute carrier family 34 member 2 (SLC34A2)	HPA037989	Sigma Aldrich	AB_10670536	Rabbit	polyclonal	1:100
Vimentin	sc-58901	Santa Cruz	AB_794004	Mouse	Vim 3B4	1:100
Cluster of differentiation 31 (CD31)	MA3100	Thermo Fisher Scientific	AB_223516	Mouse	HEC7	1:100
Anti-Mouse IgG (H+L) FITC Conjugated	62-6511	Thermo Fisher Scientific	AB_88368	Goat	polyclonal	1:200
Goat Anti-Rabbit IgG (H+L) Texas Red Conjugated	T2767	Molecular Probes	AB_221655	Goat	polyclonal	1:200

For histology, fixed membranes were dehydrated sequentially in 70, 90 and 100 % ethanol, for 30 minutes at each concentration. Inserts were then incubated in 100 % isopropanol for 30 minutes, before transfer to molten paraffin wax (65°C) for 1 hour. The membrane was then excised from the insert using a scalpel blade and embedded in paraffin, cured and sectioned at 4 μm. Sections were subjected to dewaxing and H&E staining as outlined in Lee *et al*. ^30^. Stained sections were dehydrated and mounted in DPX mounting medium (Sigma Aldrich) with a cover-slip overlaid for analysis on a Nikon Eclipse Ci upright microscope.

### Statistics

All statistical analysis was performed using GraphPad Prism version 7.03 for Windows, GraphPad Software, La Jolla, California, USA, www.graphpad.com. Replicate numbers and number of experiments are detailed in figure legends. Dextran blue measurements ( Figure 7A–B) were evaluated using a one-way ANOVA and Tukey’s multiple comparisons tests. These are presented as means ± standard deviation, where n represents individual inserts, selected at random by the researcher and assigned into groups (not blind). Statistical significance is denoted as * p ≤ 0.05, ** p ≤ 0.01, *** p ≤ 0.001 and **** p ≤ 0.0001 (Tukey’s multiple comparisons test shown). Sample size was limited by plate size and practical considerations (handling of multiple samples of this nature). We therefore performed three repeats of each experiment (n = 3 inserts per group) on different days, using fresh cells and inserts to ensure replicability of findings. To increase the statistical power of each test, data from identical groups of each day was first analysed using ANOVA and a Tukey’s multiple comparison’s test for variability, before combining replicates from each day to give a total of 9 replicates per group. Gaussian distribution was assumed for ANOVA; however, sphericity (equal variability of differences) was not and thus, the Geisser-Greenhouse correction was used.

TEER analysis was performed for the average of three readings per insert at each time point, with six inserts per group, per experiment (BATII and B2AE co-cultures). Data shown is combined from three experiments performed on different days and presented as means ± standard deviation, analysed using an unpaired t-test.

### Protocol

Here we describe the step by step procedure used to set up and validate a 14-day co-culture of BATII (ATII-derived) epithelial cells with BPAEC endothelial cells on Transwell inserts. A simplified schematic of the procedure, including timeline, is found in Figure 1, whilst all reagents may be found, with their provenance, in Table 1. In principle, the same protocol also applies to the pairing of B2AE cells in a co-culture with BPAECs; however, it should be noted that the authors have not optimised this arrangement, having chosen to focus on BATII cells for reasons outlined in the discussion.

***Step 1: Revive BPAEC cells (Day -9)***. All stages of culture are carried out in a humidified incubator, at 37°C, 5 % CO _2_.

We use cells between passages 2 and 10, for consistency and to eliminate the potential for phenotypic drift at higher passages. Cells, frozen down at 1 × 10 ^6^/mL/vial in Recovery™ Cell Freezing Medium (12648010, Invitrogen), were revived from liquid nitrogen, seeded at a density of 2 × 10 ^6^ cells per T75 cm ^2^ flask in EGM-2 (CC-3162, Lonza, UK) and cultured to 80 % confluence, without antibiotics*. EGM-2 media (500 mL) should be made up on the day of revival from liquid nitrogen, using freshly thawed Lonza bullet kit Singlequots™. After a period of one month, any remaining media of the batch should be discarded and fresh media prepared. One bottle is sufficient to maintain BPAEC cells (using 10 mL per T75 cm ^2^ flask) and also feed one 24 well plate of co-cultures for a total of 21 days (including the initial culture phase, consisting of BPAEC cells only). Medium is exchanged the day after seeding (day -8) and at 2 days (day -6), always ensuring that the cells have media exchanged the day before trypsinisation.

*These cells are not sensitive to penicillin and streptomycin; however, our downstream applications require the co-culture system to be antibiotic free.

***Step 2: Seed BPAECs onto Transwells (Day -5)***. BPAEC cells are trypsinised using 3 mL of 0.25 % trypsin/0.05 % EDTA (25200056, Life Technologies, UK) per T75 cm ^2^ flask and neutralised using an equivalent volume of DMEM/10 % FBS. The suspension is triturated to ensure homogeneity of the suspension, centrifuged at 300 × *g* for 5 minutes, then resuspended in 1 mL EGM-2 for counting, using the TC-20 automated cell counter. This takes the form of a viability count, mixing 10 μL of cell suspension with 10 μL of 0.4 % trypan blue (15250061, Life technologies). BPAEC cells are then seeded onto the apical surface of 6.5 mm diameter (0.33 cm ^2^) Transwell-CLEAR™ 8μm pore size permeable membranes in a 24 well plate, at a density of 2 × 10 ^4^ cells/insert, in 100 μL EGM-2 (approximately 6.5 × 10 ^4^ cells/cm ^2^). EGM-2 (600 μL, the volume recommended by Corning) is added to the basolateral chamber of each well. BPAECs are cultured on the Transwell inserts for 5–7 days, replacing EGM-2 in the basolateral chamber at 2-day intervals and removing apical medium which seeps through from the basolateral side of the membrane. BPAECs do not form sufficient tight junctions to prevent media seepage – this step is simply to remove old media from the apical surface.

***Step 3: Revival of BATII cells (Day -3)***. The BATII cell line has thus far not been karyotyped beyond passage 23, therefore we have restricted use between p14 and p23 in our own experiments. We freeze down BATII cells at a density of 1 × 10 ^6^ cells/mL/vial in Recovery™ Cell Freezing Medium (as before). BATII cells should be revived 3 days prior to seeding onto Transwell inserts and cultured in SAGM (CC-3118, Lonza) without antibiotics, seeding 1×10 ^6^ cells per T75 cm2 flask, to allow for the relatively high proliferation rate of these cells compared to BPAECs. As with EGM-2, SAGM should be prepared fresh using the Lonza Singlequots™ on the day of revival and any media remaining after one month discarded. BATII cells are fed the day after seeding into flasks and also the day before trypsinisation, at a volume of 10 mL per T72 cm ^2^ flask.

***Step 4: Seeding of BATII cells onto the BPAEC layer (Day 0)***. On the day of seeding, cells are trypsinised and neutralised as for BPAECs, resuspended in 1 mL EGM-2 and counted, as before. A dilution is prepared in EGM-2, at a density of 2 × 10 ^5^ cells/mL. Seeding on top of the BPAEC layer is performed at a density of 2 × 10 ^4^ cells/insert. The co-cultures are then returned to the incubator for 2 hours to allow for attachment, after which the apical medium should be removed and the cells cultured at air-liquid interface (ALI) for 14 days. Cultures achieve ALI (defined as less than 25 % of the insert submerged by EGM-2) from around 5 days after the seeding of BATII cells.

***Step 5: Feeding and TEER monitoring (Day 2 – 14)***. Cultures are maintained in a humidified incubator, at 37°C, 5 % CO _2_, feeding every 2–3 days in the basolateral compartment only (600 μL) and removing any media on the apical surface.

*Technical tip*: Media removal or addition is performed by tilting the plate to a 30° angle and placing the tip edge against the side of the well. Additions are performed slowly. This helps the user to avoid touching the cell layers and minimises cell stress.

TEER studies are carried out from 2 days after the seeding of BATII cells onto BPAEC cells in Transwells, i.e. the generation of a co-culture. BPAECs do not form sufficient tight junctions compared to a co-culture and this this is reflected in the TEER values, when comparing co-cultures or BATII monolayers to BPAEC monolayers. Measurements are acquired using the EVOM2 epithelial Voltohmmeter, with a 4 mm STX2 chopstick electrode (300523, World Precision Instruments Inc., US). When monitoring trans-epithelial electrical resistance (TEER), on the day of feeding, fresh EGM-2 should first be placed in the apical chamber (500 μL) as well as the basolateral compartment (600 μL). A blank Transwell insert is set up in another well for comparison. The nature of TEER measurement means that drift is to be expected, particularly as the plate cools down during measurement, therefore the medium should be equilibrated to temperature *in situ* (at least 15 minutes) before measurements are taken. Ideally, this temperature is 37°C; however, in practice, this is difficult to achieve if a full 24 well plate is to be measured. We therefore perform 3 measurements in each well (moving from well 1 to 24, then repeating the process twice, generating three measurements in total and using their arithmetic mean), using chopstick electrodes which are first sterilised in 70 % ethanol and rinsed in DPBS. Net TEER is calculated by subtracting the reference (blank Transwell insert only) from the mean (n=3) measured TEER of the co-culture or mono-culture sample ( Equation 2). This is then converted to Final TEER by correcting the value for the area of cell growth ( Equation 3).

Apical medium is removed after TEER measurements, to maintain ALI. Average TEER is plotted for each well at a particular time point. As part of our characterisation studies, the ‘average of averages’ (average of 3 readings taken in each replicate well, n=3 Transwells per timepoint) for each time point were plotted between days 2 and 14 of culture, with standard deviation.

*Technical tip*: to keep readings as consistent as possible, hold the chopstick electrodes in the same place for each insert in a perpendicular fashion to the plate surface, with the shorter electrode in the apical compartment and both electrodes submerged. Avoid touching the co-culture itself, or walls of the insert, as this risks damaging the cells and generating inconsistent readings. This is even more important when using 12 mm (1.12 cm ^2^) diameter inserts, as variation is greater. If readings are inconsistent for a particular well, or the resistance is unexpectedly high, consider using fine grade sandpaper to carefully rub the silver tip of the electrodes (the bulbous, inward facing area of the chopsticks). No other part of the electrode should be touched – the chopsticks are incredibly delicate, so store carefully and avoid creasing the wire.

NetTEER(Ω)=MeasuredTEER(Ω)−ReferenceTEER(Ω)(Equation 2)

$Final\;TEER\;(\Omega /cm^{\text{2}})=\;Net\;TEER\;(\Omega )\times \;Area\;of\;Transwell\;insert\;(cm^{\text{2}})\;(\text{Equation 3})$

***Step 6: Dextran Blue Studies (Day 14)***. Dextran blue permeability may be performed either at a single time point, as a measure of layer integrity, at multiple time points using single measurements, or at multiple time points sampling from the same well, enabling the calculation of ‘apparent permeability’. In the current studies, we have been comparing co-cultures with single layers of both BPAEC and BATII, therefore we have chosen to use single sampling, single time point analysis. Unlike TEER, dextran blue studies mean sacrificing the insert used; therefore, this should be allowed for when calculating replicates and so on for co-culture studies.

A 10 mg/mL stock solution should be prepared using Dextran Blue 2000 (DB2000; D5751, Sigma, MI, USA) in DPBS, alongside standard solutions of 7.5, 5.0, 3.75, 2.5, 1.25, 0.625, 0.3125 and 0.156 mg/mL. Add DPBS (600 μL) to each well of a fresh 24 well plate and equilibrate the plate to 37°C (minimum 15 minutes).

Remove inserts containing cells one by one from the original plate, removing any apical medium during the transfer. Add the dextran blue stock solution (100 μL of 10 mg/mL) to the apical surface of each insert during the transfer and place the insert in the fresh plate. A blank insert is set up alongside the co-culture/mono-cultures and used as a no cell control, along with a well containing 600 μL of DPBS and 100 μL dextran blue solution at 10 mg/mL, to determine extent of resistance exerted by the membrane.

*Technical tip:* Use two 200 μL pipettes when removing medium and adding dextran blue solution. This enables the user to have one pipette set to 100 μL (for dextran blue) and the other to 200 μL, to ensure that all apical medium is removed (if present – co-cultures should have very little at 14 days).

Return the plate to 37°C, 5 % CO _2_ for 1 hour. At the end of the incubation, remove 600 μL from the basolateral compartment and read at 630 nm on a spectrophotometer in a 1 mL volume disposable cuvette. This should be carried out alongside 600 μL of the prepared standards. To allow for lower volumes in the apical compartments, we dilute an aliquot (60 μL) from apical solutions in 540 μL DPBS (a ten-fold dilution), before reading at 630 nm. Unknown values may then be extrapolated from a linear regression of the standard curve and corrected for dilutions accordingly.

***Step 7: IF Microscopy of whole inserts (Day 14 onwards)***. Cells cultured for 14 days are used for IF studies, to ensure that structural features reminiscent of the native alveolar epithelium are present, in particular a pseudostratified mix of ATII and their differentiated counterparts, ATI. At this stage, the endothelial layer of BPAECs have started to migrate through the membrane pores. This allows a more consistent contact with the underlying medium and also has the effect of providing ‘anchorage’. For this reason, we do not use an ECM to coat our Transwells, although we have included the protocol in our Methods, for users who wish to culture ATII cells or cell lines as a monolayer.

*Technical tips:* It is possible to divide membranes to maximise output; however, in our experience, the medial area of the insert is very delicate, so caution should be exercised and a fresh scalpel or razor blade used for each membrane, rocking the blade across the membrane rather than slicing, to reduce shear forces. When probing whole inserts, this may be done *in situ*, adding 100 μL of antibody solution to the apical side and 50 μL to the underside, holding the tip against the outer edge of the vertical wall of the insert and allowing the solution to be drawn under the membrane by capillary action.

Transwell membranes are first washed three times (gently, holding the pipette against the inner wall of the insert when washing the apical surface) with DPBS. Cultures are then fixed in 4 % PFA for 15 – 30 minutes at room temperature. Each membrane is then washed three times in immunofluorescence buffer (IF buffer, details in Table 2), 5 minutes per wash. This also permeabilises the cells.

Permeabilised cultures are blocked in DPBS/ 5 % normal goat serum (S26-M, Sigma Aldrich)/ 0.1 % triton X-100 (X100, Sigma Aldrich) for 1 hour, at room temperature. All antibody solutions are then made up in the same blocking buffer and applied to the apical and basolateral aspects of the membrane (see tips above). The plate containing the inserts are then incubated overnight at 4°C. The next day, inserts are washed three times with IF buffer (5 minutes per wash at room temperature) and secondary antibody applied for 1 hour at room temperature in the dark. Secondary antibodies are applied as follows: cells are again washed three times with IF buffer (5 minutes per wash at room temperature) and the membrane removed from the insert, using a fresh scalpel blade. Resting the inverted insert on a flat surface, run the leading edge of the blade around the circumference of the insert, turning the insert to complete the circuit, rather than moving the blade. This minimises ruffling of the membrane and serration of the membrane edge. The membrane is then placed onto a coverslip for mounting, apical side up. Place one drop of Prolong® Gold Antifade reagent with 4′, 6-diamidino-2-phenylindole (DAPI) (P36941, Thermo Fisher Scientific, Waltham, MA) or Vectashield Hardset mounting medium with phalloidin (TRITC) (H-1600, Vector Laboratories, Burlingame, CA, US) onto the centre of the membrane. Overlay the sample with a second clean coverslip, taking care not to introduce bubbles. Imaging may be performed after curing, using a confocal microscope. Primary antibodies are detailed in Table 4.

Note: The Transwell insert membrane does auto-fluoresce in the near UV excitation spectrum, therefore may exert interference with DAPI staining, in the form of strong background signal. If this occurs, another nuclear stain should be used if required.

***Step 8: Fixation, embedding and haematoxylin & eosin (H&E) staining (Day 14 onwards)***. Inserts need to be carefully treated due to the delicate nature of co-cultures. For this reason, all fixation, embedding and staining procedures are performed manually. Fix the inserts by immersion under 1 mL 10 % Formalin, divided between the apical and basolateral compartments. Avoid fixing for greater than 24 hours since tissue antigens may either be masked or destroyed. Thirty minutes is adequate for insert membranes. Because paraffin is immiscible with water, tissue must be dehydrated at room temperature, before adding molten paraffin wax. To do this, immerse in 70 % ethanol for 10 minutes, in 90 % ethanol for 10 minutes, in 100 % ethanol for 10 minutes and finally in isopropyl alcohol (IPA) 100 % for 5 minutes.

*Technical tip*: Using IPA instead of xylene allows the steps to be performed with the insert *in situ*, which makes handling and orientation easier.

Place the whole insert into IPA/paraffin 1:1 for up to 1 hour at 58°C. We perform this step at the embedding station, using pre-warmed IPA and pre-mixing IPA with paraffin in a vessel large enough to accommodate one insert, but small enough to sit in the molten wax compartment. After 1 hour, embed the tissue in paraffin at 58°C for another hour. At this stage, the membrane should be removed from the insert with a fresh scalpel, taking care to keep the insert apical side up in order to minimise damage to cells. Removed membranes are then embedded in fresh paraffin in a mould deep enough to accommodate them, with the thin edge perpendicular to the edge of the microtome blade. The block is allowed to cool at the embedding station, then stored at room temperature or 2–8°C until sectioning.

For best results, perform sectioning after blocks have been on ice (fill a suitable container with water and freeze at -20°C to form a solid block of ice) for 30 minutes. Cut 4–8 µm thick sections using a rotary microtome and float the sections onto a pre-heated 56°C water bath. Collect sections on histological slides (Superfrost +/+) and dry the slides overnight at 37°C. Slides can then be stored, either at room temperature or at 2–8°C. for several years in slide storage boxes.

***Haemotoxylin and Eosin (H&E) Staining***. The staining procedure is outlined in detail in Lee *et al.* ^30^, therefore this section details technical tips only.

*Technical tip 1:* All dewaxing and staining steps are carried out manually in a fume hood, since cells in a co-culture are prone to detaching during staining/wash procedures in automated systems.

*Technical tip 2*: Sections cut from embedded membranes are best cut at a minimum of 4 μm. We have also successfully stained 8 μm sections, which are less susceptible to detachment from the slide; however, this does affect clarity of images.

*Technical tip 3:* Rinsing slides under running tap water is best performed by filling a sandwich tub or similar with tap water and lowering a hose from the tap into the water, with the tap turned on to a trickle. Once confident that the water pressure is as low as possible, the user can *then* lower the cradle with slides into the tub. If this is still too aggressive and sections are detaching from the membrane or slide, we have successfully substituted running water for repeated rinsing in static tap water. To perform the rinsing step in this way, simply fill the tub with tap water and lower the cradle into the water *without* the hose. Leave for 5 minutes and gently lift the cradle out. Empty the tub and repeat the process until no stain is visible in the water (normally three to four washes).

## Results

### Immortalisation of ATII

**Figure 2.**
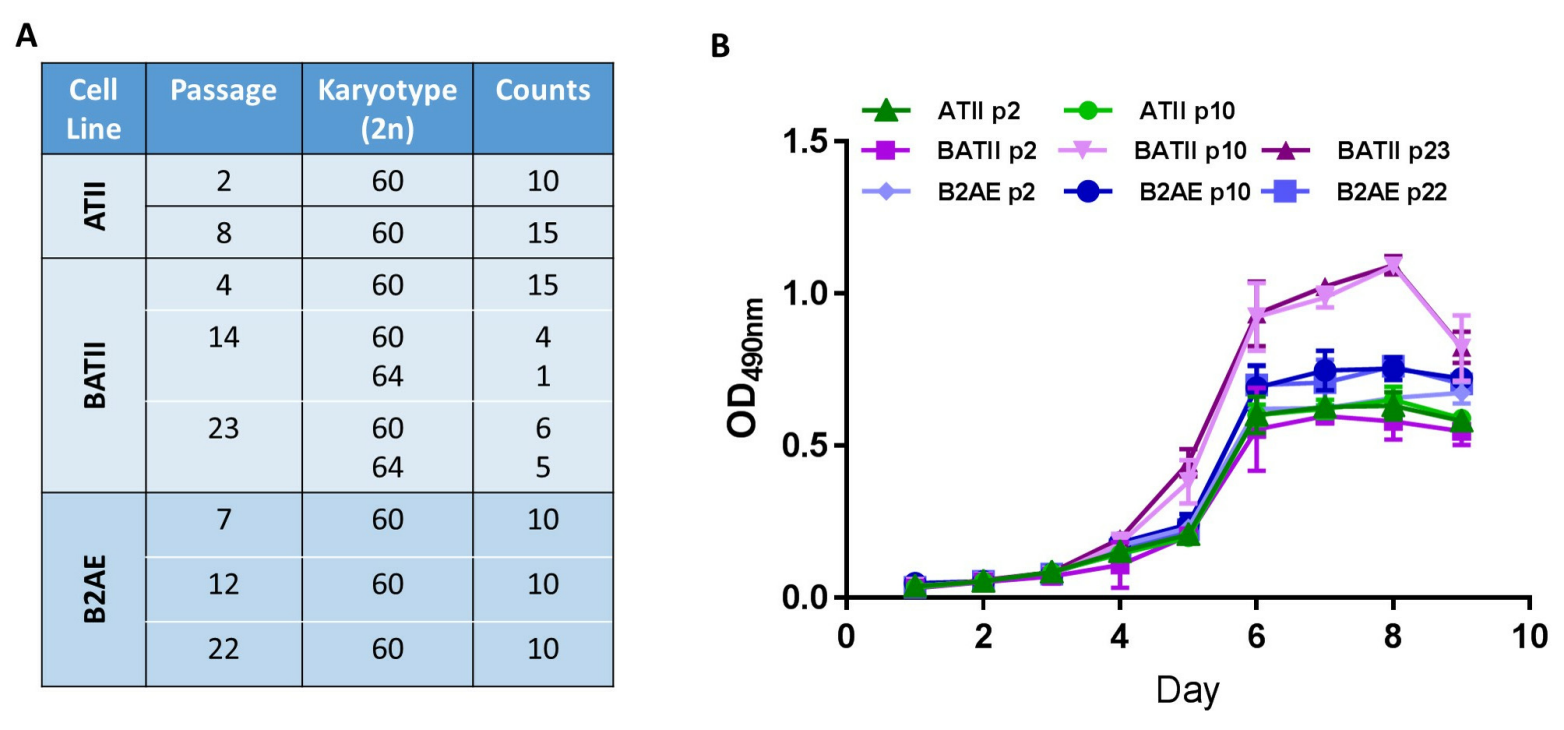
Characterisation of cell lines using karyotype and growth curve analysis. For karyotype analysis, a metaphase spread was performed on Colcemid cell cycle arrested cultures for both BATII and B2AE, comparing with the wild-type ATII ( **A**). The Bmi1 */*hTERT transduced B2AE cell line was found to maintain the wild type bovine karyotype of 2n = 60, whilst the SV40 */*hTERT transduced BATII cell line showed signs of genomic instability at both passages 14 and 23. This was reflected in a growth curve analysis of the two cell lines, which was performed by seeding primary ATII cells into a 96 well plate, comparing over 9 days with the BATII and B2AE cell lines ( **B**), using the Cell Titre Aqueous One Assay (Promega). Later passages (p10 and p23) of BATII cells exhibited a considerably higher rate of proliferation than the wild type ATII, whilst the B2AE cell line at all passages was comparable with its wild type counterpart. Data shown as optical density read at 490 nm (OD _490nm_); Mean ± SD, n=3.

We have isolated ATII cells as outlined in Lee *et al*. ^30^ ( Figure 1) and immortalised them using two gene combinations through lentiviral transduction. The generation of stable cell lines allowed us to generate more consistent results between studies within our own laboratory and between other researchers worldwide. The use of the stem cell regulator and proto-oncogene Bmi1 in conjunction with hTERT was used by Fulcher *et al*. to generate a novel human bronchial epithelial cell line ^34^ with considerable success. Like Fulcher *et al.*, we found that the Bmi1 */*hTERT transduced B2AE cell line maintained genomic stability for up to at least 22 passages ( Figure 2A). In addition, the proliferation rate was almost identical to that of the wild-type ATII cell ( Figure 2B). Conversely, the SV40 */*hTERT transduced BATII cell line shows signs of genomic instability ( Figure 2A) characteristic of viral oncogene derived cell lines ^35, 36^ at later passages, with a corresponding increase in proliferation rate by passage 10 ( Figure 2B).

**Figure 3.**
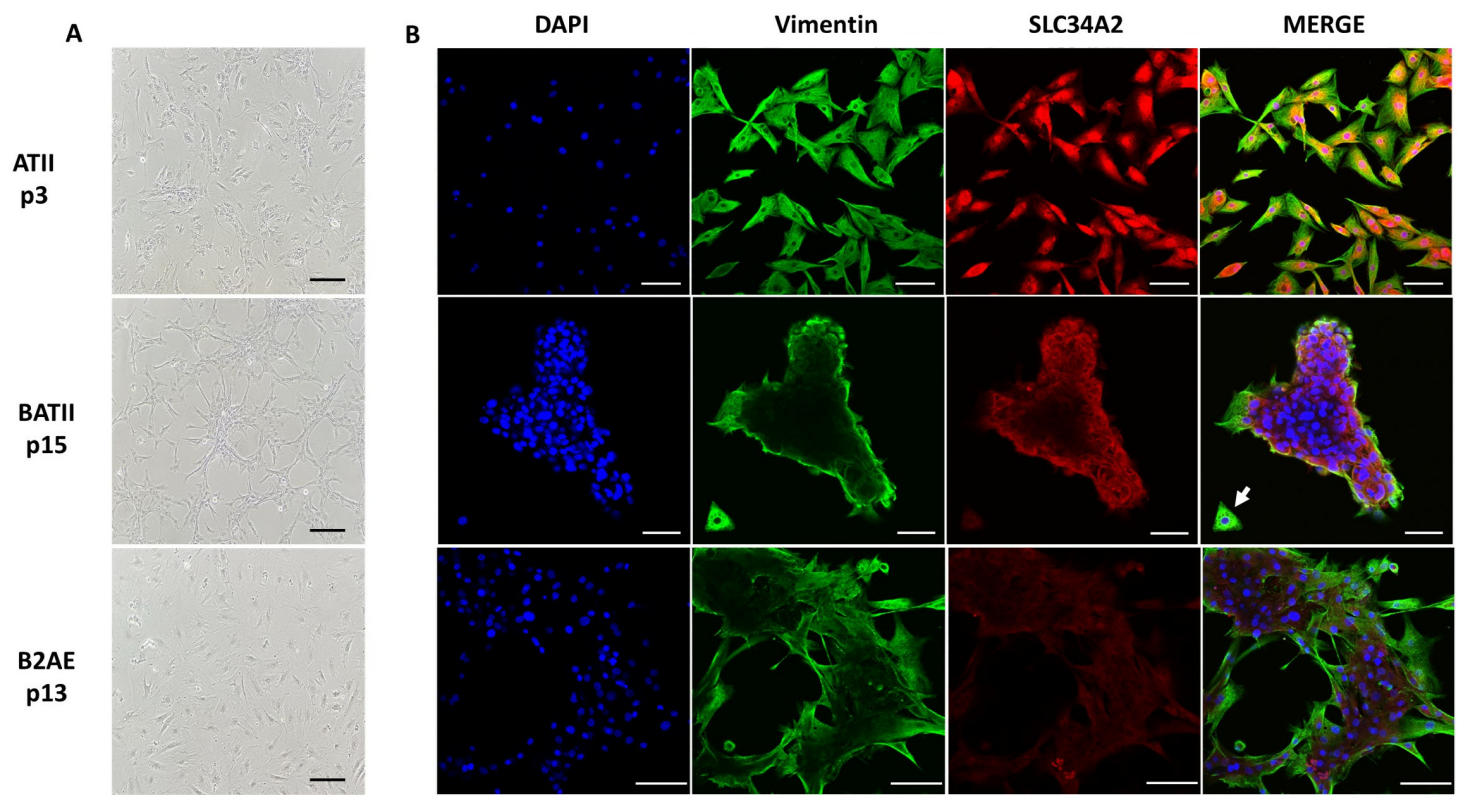
Characterisation of BATII and B2AE cell lines using light microscopy and confocal imaging of alveolar type II cell markers. Light microscopy images of the wild type ATII, SV40 */*hTERT lentivirus transduced BATII and Bmi1 */*hTERT lentivirus transduced B2AE were generated from cultures seeded into T75 cm ^2^ flasks and imaged on a Nikon Eclipse Ci upright microscope ( **A**). Scale bar 50 μm. For IF, each cell type was seeded onto a Nunc Lab Tek II coverglass, before fixation and staining with the ATII marker SLC434A2 and vimentin, a marker of epithelial-mesenchymal transition ( **B**). Both cell lines were more prone to colony formation than their wild-type counterpart, with BATII cells in particular forming 3D structures, whilst B2AE frequently formed ring-like colonies around a central ‘lumen’. Cells which exhibited a flattened morphology on the exterior of the colonies of both cell lines stained most strongly for vimentin, with one lone cell in the BATII image notable for the complete absence of SLC34A2 (middle panel, white arrow), which remained strong in the centre of the 3D colony. Scale bar = 100 μm.

We analysed monolayers of each cell line alongside the wild-type ATII, as cultures seeded into T75 flasks and onto Lab-Tek II coverglass. Whilst cells were more evenly distributed when cultured on plastic (T75 flasks) ( Figure 3A), both BATII and B2AE cells formed colonies on coverglass ( Figure 3B); however, the BATII cells in particular formed three-dimensional structures ( Figure 3B, middle row). These were flatter on the periphery and raised in the centre. The flattened morphology on the outside edge of the BATII colonies corresponded to a strong signal for the mesenchymal marker vimentin, the expression of which has been previously reported as being instrumental to the role of ATII progenitor cells in wound healing ^37^ and indicative of the upregulation of mesenchymal markers in ATII cells cultured under two-dimensional conditions ^38^. The stronger staining on the periphery of BATII cells reflected that observed in the wild type ATII ( Figure 3B, top row) and was also observed in the B2AE cell line ( Figure 3B, bottom row). The phosphate transporter SLC34A2 is expressed exclusively in the ATII cell in lung tissue and is therefore considered a marker for ATII cells ^39– 41^. Whilst the ATII marker SLC34A2 was notably absent from the cells on the outside edge of the colonies seen in the BATII and B2AE cell lines, it was very much evident in the adjacent cells of the colony, which formed the inner margins.

The concomitant increase of vimentin expression and decrease in SLC34A2 expression was most notable in the isolated colony of the BATII culture ( Figure 3B, middle row, white arrow). This can be seen in more detail in the movie, presented in Dataset 1, see *Extended data* ^42^. The B2AE colonies were flatter in nature ( Figure 3B, bottom row) and thus more mesenchymal in terms of protein expression, with visibly reduced SLC34A2 expression throughout with vimentin expression that was comparable to the wild type ATII cultures. This observation, reflective of the flatter nature of the colonies in these cultures, indicates that the B2AE cell line differentiates readily to ATI, taking on the mesenchymal phenotype. Whilst these observations reflect the differentiation of ATII cells *in vivo*, our laboratory intended to use the resulting cell line for further studies of host-pathogen interaction, with particular focus on the ATII cell. This requires a cell line that proliferates more rapidly in order to generate a stock of cell, in addition to maintaining ATII characteristics; traits that were exhibited better by the BATII cell line. For this reason, further characterisation for our purposes were restricted to the BATII cell line.

### Matrigel cultures

**Figure 4.**
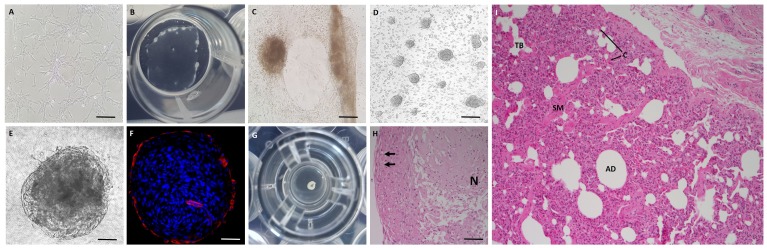
Comparison of culture surfaces and their effects on BATII phenotype. BATII cells (p11) were grown on uncoated 2D plastic ( **A**), 2D plastic coated in 10 % Matrigel ( **B**– **C**) and a 12 mm diameter (1.12 cm ^2^) Transwell insert ( **D**– **H**). When cultured on uncoated plastic, BATII cells formed colonies ( **A**). On 10 % Matrigel coated plastic, the cells formed sheets ( **B**), containing 3D spheroid structures ( **C**). When cultured on Transwell inserts, these spheroids developed into organoid like structures ( **D**– **E**). Immunofluorescence probing for the ATII marker SLC34A2 revealed that the organoid like structure was strong for this ATII marker at the periphery, whilst the ‘lumen’ was devoid of any expression ( **F**). Embedded and sectioned organoids ( **G**) were stained with haematoxylin and eosin ( **H**), which revealed a central, necrotic area, N, with live cells at the periphery (arrows). Scale bars are 50μm ( **A**), 50 μm ( **C**), 100 μm ( **D**), 25 μm ( **E**– **F**) and 50 μm ( **H**). The insert pictured in ( **C**) is 12 mm diameter (1.12 cm ^2^), with the organoid estimated at 3 mm diameter. A cross-section of H&E stained bovine distal lung is shown for comparison ( **I**), highlighting terminal bronchioles (TB), alveolar duct (AD), smooth muscle (SM) and capillaries (C).

Previous literature has explored the role of extracellular matrix in the culture of epithelial cells of the distal airways ^38, 43^. Since we intended to focus upon the use of the BATII cell line to model bovine alveolar epithelium, we explored the use of Matrigel in our early BATII cultures, coating 2D plastic and Transwell inserts with a 10 % solution of Matrigel in growth medium. Whilst cells seeded onto plastic (24 well format) formed the colonies thus far characteristic for this novel cell line ( Figure 4A), the same cells, when seeded onto plastic coated with a 10 % solution of Matrigel, formed a sheet of epithelial cells ( Figure 4B–C), which eventually peeled away from the sides of the well. These sheets contained 3D structures, a feature even more prominent when the cells were seeded onto 12 mm (1.12 cm ^2^) diameter 3 μm pore size Transwells ( Figure 4D–H). The spheroidal, cyst-like structures seen in Figure 4D were reminiscent of those observed by Lee *et al.* ^44^ and appeared to contain debris in the ‘lumen’ ( Figure 4E). The structure was found to strongly express the ATII marker SLC34A2 around the periphery, whilst expression was notably absent in the ‘lumen’ ( Figure 4F). When allowed to develop further (> 7 days), the sheet peeled back from the periphery of the Transwell insert membrane and formed a larger organoid structure visible with the naked eye ( Figure 4G). This was embedded and sectioned for H&E staining and found to contain a live periphery ( Figure 4H, black arrows) and a necrotic core ( Figure 4H, ‘N’). Whilst these cultures exhibited an ATII phenotype, they were not suitable for transport experiments, since they could not be cultured at air-liquid interface as a result of the peeling away from the Transwell edge. Extracellular matrix (ECM) is not necessary for the culture of co-cultures, since endothelial cells produce a laminin-rich ECM in culture ^45^. We therefore did not pursue the 3D Matrigel culture of the BATII cell line further.

### Co-culture characterisation (immunofluorescence)

**Figure 5.**
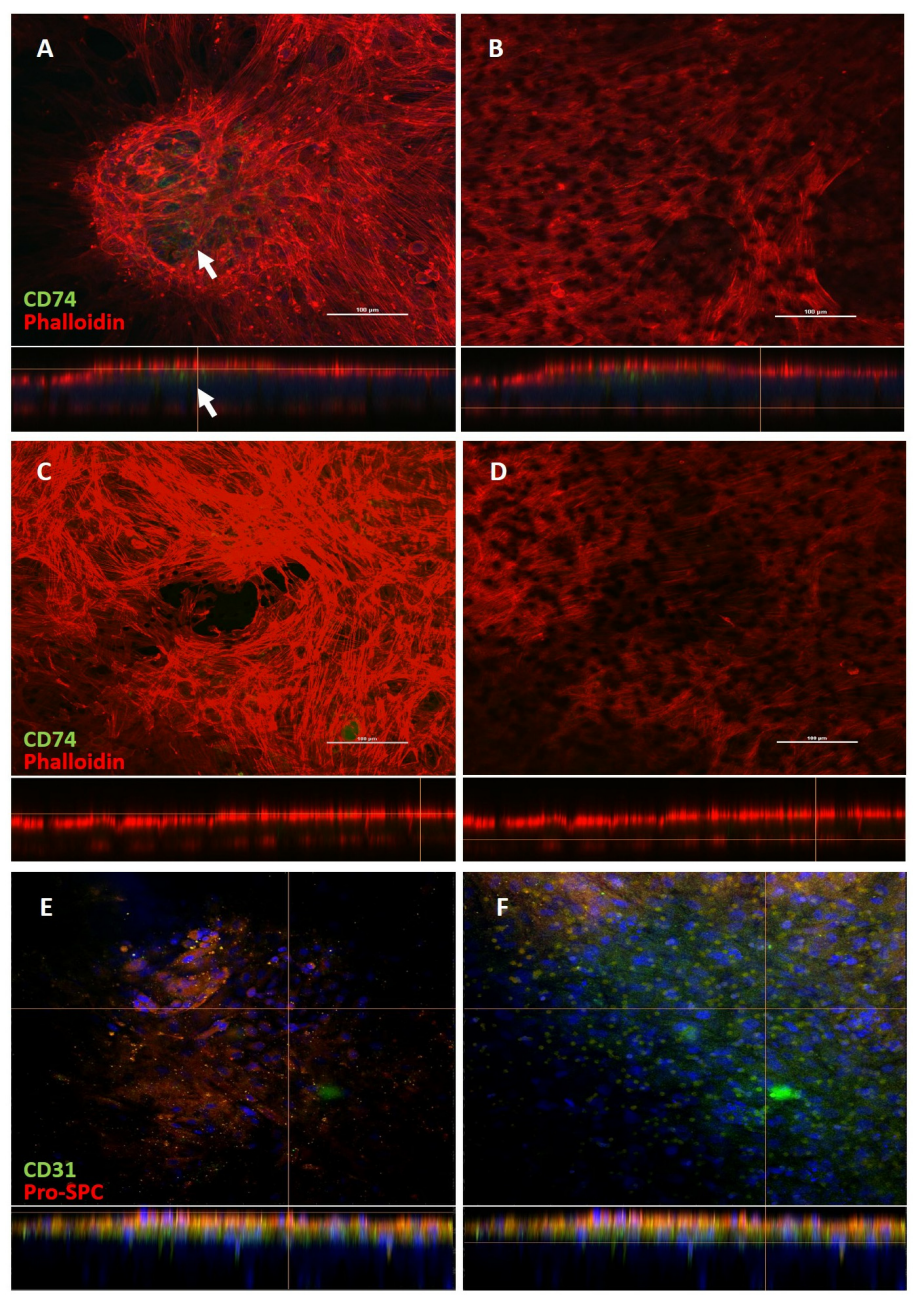
Confocal (Z-stack) analysis of BATII and B2AE epithelial cell lines, incorporated into co-culture models. Co-cultures consisting of either the BATII ( **A**) or B2AE ( **C**) epithelial cell line overlaid onto a BPAEC ( **B** and **D**) endothelial layer were cultured for 14 days at air-liquid interface (ALI) and stained for the ATII marker CD74 and phalloidin ( **A**– **D**). The B2AE co-culture was also stained for the ATII cell marker Pro-SPC and the endothelial cell marker CD31 ( **E**– **F**). BATII cells formed multiple 3D structures on the apical surface of the co-culture, the centre of which stained strongly for the ATII marker CD74 ( **A**, arrow). Conversely, BPAEC cells formed a thin and flattened layer, which partially migrated through to the basolateral aspect of the insert membrane ( **B**), the pores of which can be seen in the image and are thus used as point of reference when moving through the Z-stack. The B2AE cell line seemed to form a more uniform epithelial layer ( **C**), which stained poorly for CD74. This did not affect the morphology of the BPAEC layer ( **D**), which also migrated through the pores, to the basolateral aspect. Whilst B2AE cells do not stain strongly for CD74, they do retain the classical ATII marker, Pro-SPC ( **E**), whilst CD31 was used to highlight the endothelial BPAEC layer ( **F**). Below each image is the corresponding Z zoom (500 %) segment of the orthographic slice, including the Z slice indicator (yellow line). Scale bar = 100 μm.

Our laboratory aims to use ATII cell lines in studies of host-pathogen interactions, focussing on bovine tuberculosis infection and transport across an epithelial/endothelial co-culture. We therefore grew co-cultures of both novel ATII-derived epithelial cell lines for comparison for 14 days, before probing for the ATII surface marker cluster of differentiation CD74 and mounting in TRITC-labelled phalloidin to highlight cell morphology and co-culture thickness by F-actin visualisation. We were particularly interested in whether the B2AE cell line in ALI culture would differentiate rapidly to ATI-like cells, as we observed on coverglass. CD74 was reported by Lee *et al.* to be present on ATII cells also expressing pulmonary surfactant protein C (SP-C) ^3^ and so was chosen here as a marker. The apical aspect of the BATII co-culture ( Figure 5A) revealed 3D structures, containing cells which strongly expressed CD74 (white arrows), whilst the surrounding flatter areas only stained positive for phalloidin. This confirmed observations made under the light microscope that the multi-layered nature of the 3D structures was not universal across the membrane and that the epithelial layer was heterogeneous. The basolateral aspect ( Figure 5B) also stained strongly for F-actin and demonstrated the difference in morphology between the ATII derived BATII cells and the endothelial BPAECs. These can be observed in more detail in Dataset 2, see *Extended data* ^42^. Similar differences were observed in the phalloidin staining between the B2AE apical ( Figure 5C) and basolateral ( Figure 5D) aspects. The B2AE cell line did not appear to form 3D structures and CD74 expression appeared to be absent in these co-cultures. Whilst B2AE appear to lose the ability to express CD74 when cultured at ALI, they do retain the ability to express the ATII marker surfactant protein C ( Figure 5E), a finding which reflects the heterogeneity of ATII cells and the phenotypic plasticity of the ATI cell ^46^. The endothelial surface marker CD31 was used to distinguish the BPAEC endothelial layer from the B2AE epithelial layer ( Figure 5F); however, the poor signal and flattened morphology rendered it difficult to distinguish this cell line from the epithelial layer using endothelial specific markers. To summarise, the B2AE cell line loses the ATII specific marker CD74, whereas the BATII cell line does not. Together with the relatively slow proliferation rate, we conclude that the B2AE cell line is less amenable to modelling the bovine alveolar epithelium than its BATII counterpart in these respects.

### Co-culture morphology

**Figure 6.**
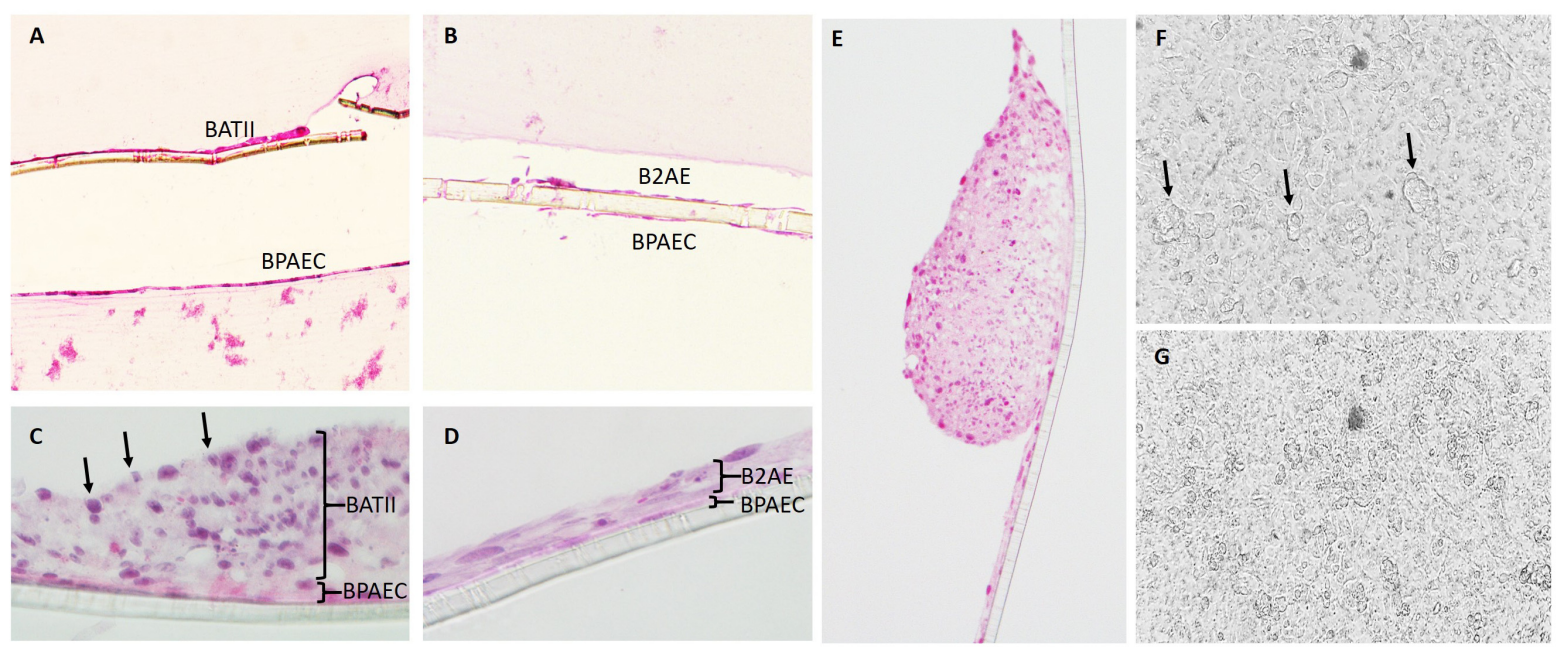
Characterisation of co-cultures using histological analysis (haemotoxylin and eosin) and light microscopy. Co-cultures containing both cell lines were also stained for morphological analysis using haematoxylin and eosin. An apical and basolateral layer is clearly observed in both the BATII ( **A**) and B2AE (magnification 10 x) ( **B**) co-cultures (magnification 20 x). The fragility of the membrane during processing meant that it was difficult to visualise the intact co-culture and so a co-culture was seeded onto a Transwell 12 mm diameter (1.12 cm ^2^) insert with 0.4 μm pore size. This reduced the likelihood of cell detachment, as the endothelial cells remained on the apical aspect of the membrane, resulting in images where the epithelial BATII ( **C**) and B2AE ( **D**) layers are distinguishable from the underlying BPAEC, in which the staining is less intense (magnification 60 x). Cuboidal cells are visible particularly on the apical surface of the BATII co-culture ( **C**, arrows), whilst the B2AE appear to be flatter and more reminiscent of the squamous alveolar type I (ATI) cells. Whilst numerous, the multi-layered regions of BATII cells did not entirely cover the surface of the endothelial BPAEC layer ( **E**), being surrounded by a thinner monolayer/bilayer of epithelial cells. Visualisation by light microscopy of the BATII/BPAEC co-culture shows the formation of the BATII epithelial layer on the apical surface ( **F**); examples of raised areas denoted by arrows, in contrast to the underlying endothelial BPAEC monolayer ( **G**) (magnification 20 x).

We used haematoxylin and eosin (H&E) staining to study the morphology of the co-cultures in 4 μm cross-sections taken from paraffin embedded Transwell inserts ( Figure 6). Images clearly demonstrate the presence of two distinguishable layers on the apical and basolateral aspects of the membrane in both BATII ( Figure 6A) and B2AE ( Figure 6B) co-cultures; however, detachment from the membrane during H&E processing was an issue and consequently, high-resolution images could not be obtained due to the distance between detached layers and the membrane. A repeat of the staining on sections acquired from co-cultures seeded onto Transwell 12 mm diameter (1.12 cm ^2^) inserts with 0.4 μm pores enabled the visualisation of the cuboidal BATII cells ( Figure 6C) as a distinct layer from the underlying endothelial BPAECs, whilst the B2AE exhibited a more flattened morphology ( Figure 6D), characteristic of the ATII descendants, the alveolar type I (ATI).

Using light microscopy, it was possible to visualise the 3D structures developed by the BATII epithelial layer when grown as part of a co-culture ( Figure 6E-F). These were not continuous across the whole culture, forming ‘islands’ in the epithelial layer. This was in contrast to the underlying BPAEC, which formed a flattened monolayer ( Figure 6G). We attribute these differences to the increased propensity of the B2AE cell line to differentiate into ATI cells under our culture conditions and suggest that the BATII cell line’s increased proliferation rate correlates with a maintenance of the ATII phenotype, in accordance with the role of ATII as a progenitor cell ^5^

### Co-culture integrity

**Figure 7.**
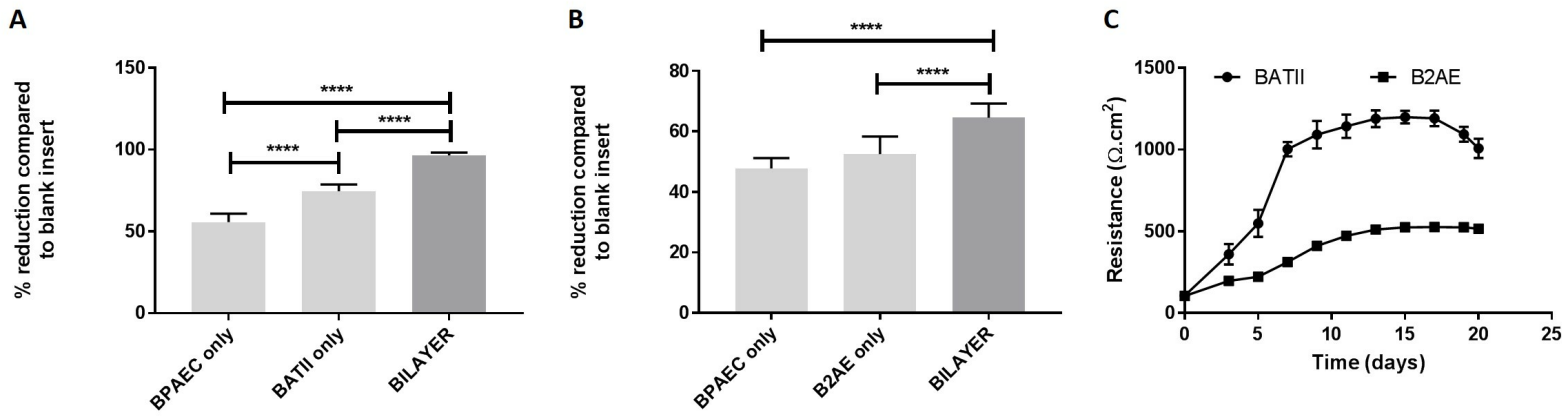
Dextran blue permeability and trans-epithelial electrical resistance (TEER) as a measure of co-culture integrity. The integrity of co-cultures containing BATII ( **A**) and B2AE ( **B**) epithelial cells overlaid onto BPAEC endothelial cells were measured by dextran blue 2000 (DB2000) permeability, following 14 days culture at air-liquid interface (ALI) on 6.5 mm diameter (0.33 cm ^2^), 8 μm pore size Transwell membranes. Co-cultures incorporating BATII cells as the epithelial layer significantly reduced apical to basolateral transport of DB2000 by almost 100 % (ANOVA, with Tukey’s Multiple Comparisons test; **** P ≤ 0.0001), whilst B2AE co-cultures reduced permeability by 65 % (**** P ≤ 0.0001). Whilst this was significant compared with inserts seeded with BPAEC cells only, this was a comparatively lower effect. Data shown is combined from three experiments (Mean ± SD; n= 9 inserts per group) performed on different days. The increased permeability of DB2000 through B2AE co-cultures was reflected in the trans-epithelial electrical resistance (TEER) ( **C**). Readings were significantly lower for B2AE co-cultures from day 3 (unpaired t-test, P ≤ 0.0001). Combined data shown from three experiments performed on different days, displayed as Mean ± SD; n=18 (inserts per group).

Using the naked eye, it was possible to visualise the presence of any medium which had seeped through from the basolateral chamber to the apical side of the membrane. The BATII-containing co-cultures were at air-liquid interface (ALI) from around 7 days, with less than 20 μL medium present in the apical chamber on feeding days. Further evidence of integral tight junction formation was generated in the significant reduction of dextran blue 2000 (DB2000) permeability across co-cultures seeded onto 6.5 mm diameter (0.33 cm ^2^) Transwell inserts ( Figure 7A and Dataset 3, see *Underlying data* ^42^); this was an average (mean) of 96 % for the BATII derived co-culture (assigning blank insert as 0 % reduction), compared to 75 % reduction imparted by the BATII only monolayer (Tukey’s multiple comparisons test; P ≤ 0.0001) and 56 % for the endothelial BPAECs (P ≤ 0.0001). This was in stark contrast to the B2AE derived co-culture, which reduced DB2000 permeability by 65 %, compared to a 53 % reduction by the B2AE only monolayer ( Figure 7B) (P ≤ 0.0001). There was no significant difference between the B2AE and BPAEC monolayers, the latter of which reduced DB2000 permeability by 48 %. This suggests B2AE cells formed a less integral layer than BATII cells, supported by the considerably lower TEER values of B2AE derived co-cultures, when compared to BATII derived co-cultures, obtained over 21 days after seeding the epithelial layer ( Figure 7C and Dataset 4, see *Underlying data* ^42^). This finding was significant (unpaired t-test; P ≤ 0.0001). We also compared measurements taken from each pore size (0.4, 3.0 and 8.0 μm). No significant differences were found (One way ANOVA, followed by Tukey’s multiple comparisons test).

## Discussion

We describe here a method by which researchers may construct a co-culture model for medium term culture, consisting of an immortalised alveolar type II (using the BATII novel cell line) epithelial cell layer overlaid onto a bovine pulmonary arterial endothelial cell (BPAEC) layer. Together, these two cell types recapitulate the highly gas permeable co-culture observed *in vivo* in the bovine alveolus. This is the first bovine co-culture model of the alveolus presented in the literature to our knowledge and thus has particular value for the progression of research into bovine respiratory diseases, zoonoses and host-pathogen interactions of the distal airways.

In order to reduce inter-experimental variability and therefore provide consistent data that are comparable with other studies, we opted to immortalise the ATII cells that were isolated according to our previously published protocol ^30^. Conventionally, immortalisation involves the introduction of viral oncogenes, for example those encoding the E6 and E7 proteins of human papilloma virus (HPVE6/E7) and the large tumour (T) antigen derived from Simian virus (SV40). These, however, result in the trade off of genomic instability with the increased proliferation rate, which arises through the loss of the p53 checkpoint ^48^, amongst other notable gene expression anomalies and an aberrant karyotype ^35, 36, 49^. For this reason, we chose to generate an additional cell line (characterising the two lines concurrently), combining the proto-oncogene and stem cell regulator B cell-specific Moloney murine leukemia virus integration site 1, Bmi1 with the catalytic subunit of human telomerase, hTERT, to enable comparison with an SV40/hTERT immortalised cell line. The introduction of hTERT alone extends proliferative capacity; however, it does not fully immortalise cells ^50, 51^. Conversely, the combination of hTERT with Bmi1 has been previously reported to result in a genetically stable human bronchial epithelial cell (HBEC) line ^34^, with the ability to form a differentiated, pseudostratified epithelium when grown at air-liquid interface (ALI), with a similar proliferation rate to non-immortalised cells. More recently, this combination was also used to successfully generate a human alveolar type II cell line ^52^. Other alternatives to viral oncogenes include cyclin-dependant kinase 4 ( *Cdk4*), as used by Ramirez *et al*. ^50^. In their characterisation of HBECs immortalised using the combination of hTERT and *Cdk4*, Ramirez *et al.* do report regions of duplication (chromosomes 5 and 20). The gene expression profile, however, closely aligns with the wild-type and therefore this combination may yield another useful cell line derived from ATII cells.

In our studies, the combination of Bmi1 and hTERT in the generation of the B2AE cell line by lentiviral transduction did indeed yield a proliferation rate comparable to ATII cells and a stable karyotype. However, our intentions to use the cells in a medium-throughput assay to study host-pathogen interactions were hampered by the slow growth. This, together with the readiness of the B2AE to develop a squamous morphology indicative of differentiated ATI cells on plastic and when cultured as part of a co-culture, made this cell line less suitable for studies of the role of ATII cells in innate immunity under ALI growth conditions.

Continuous epithelial cell lines are often reduced in their differentiation capacity (for example the BEAS-2B line ^53^), sometimes do not form the polarised layers characteristic of epithelial cells (for example the 16HBE14 ^0-^ line ^54^) or demonstrate a reduced ability to form tight junctions (for example the A549 line) ^55^. It was therefore important to characterise both the BATII and B2AE cell lines for these traits. We found non-specific staining with the zonula occludens (ZO-1) antibodies tested in bovine cultures and thus did not include these data. Instead, the use of dextran blue 2000 kDa (DB2000) permeability was employed to complement transepithelial electrical resistance (TEER). The obvious disadvantage of DB2000 is that downstream processing is extremely limited, effectively sacrificing replicates. On the other hand, TEER may be used to monitor cultures over time non-invasively, provided that care is taken with regards to positioning of the electrodes in order to avoid damage to the cell layer and minimise variance in measurements. Whereas TEER measurements reflect the ionic conductance of the paracellular pathway in the culture, the flux of non-electrolyte tracers such as dextran blue are used to indicate paracellular water flow ^56^, as well as the pore size of the tight junctions when measured over time (expressed as permeability coefficient). In the current study, dextran blue was used in the manner of Bermudez *et al.* ^57^ and Birkness *et al.*, ^33^ as a single time point indicator of co-culture integrity.

The B2AE cell line showed a reduced propensity to develop tight junctions, as indicated by the formation of a relatively inefficient barrier to DB2000 when compared to the BATII cell line and relatively low TEER measurements over 21 days, when cultured as the epithelial component of a co-culture on Transwell inserts ( Figure 7C and Dataset 4, see *Underlying data* ^42^). A recently reported human alveolar epithelial cell line generated by the introduction of Bmi1 and hTERT ^52^ reported TEER values of up to 400 Ω.cm ^2^. Our own findings were that the B2AE cell line generated a maximum TEER of circa 500 Ω.cm ^2^ by day 10 of co-culture with the endothelial BPAEC line. This was insufficient to maintain ALI beyond a couple of hours; therefore, the majority of culture time of B2AE co-cultures was as a submerged culture. By contrast, the BATII cell line reached values in excess of 1200 Ω.cm ^2^ in both mono and co-culture models. These values were comparable to bovine primary ATII (Dataset 5 and 7, see *Underlying data*), rat primary alveolar epithelial cells and considerably higher than the human ATI-like A549 line ^58^. BPAEC cells alone did not generate TEER values of greater than 200 Ω.cm ^2^, suggesting they contribute relatively little to the impermeability of the co-culture. We also examined the relationship between TEER and insert pore size in the co-culture of BATII and BPAEC at ALI, comparing 0.4, 3.0 and 8.0 μm pore sizes. Pore size appeared irrelevant to co-culture integrity, as evidenced by the lack of variation in TEER between groups (Dataset 5 and 7, see *Underlying data*). As our intention is to use the model in migration studies of mycobacteria and peripheral blood mononuclear cells (PBMCs), it was necessary to characterise the co-culture model using 3.0 and 8.0 mm pore sizes to ensure the membrane would not impede migration of bacteria and cells. However, these larger pores made histology particularly difficult. We therefore included co-cultures seeded onto 0.4 μm inserts in our characterisation ( Figure 6), which reduced the detachment of cells.

It is possible that in our studies, the repression of p53 by the large T antigen of SV40 ^48^ has had a serendipitous beneficial effect. Certainly, when cultured at ALI in the presence or absence of BPAECs, the BATII cell line retains the ATII markers CD74 and SLC34A2 to a much greater degree than the B2AE cell line. Previous studies have reported that CD74 is lost quickly in the culture of primary ATII cells as they differentiate into ATI ^2, 59^. The BATII cell line appeared to retain expression of CD74 after two weeks culture at ALI. It has been proposed that CD74 is a marker of proliferative capacity in ATII cells and that expression of this surface marker enables interaction of ATII cells with the macrophage migration inhibitory factor (MIF), stimulating ATII proliferation and alveolar repair in response to the loss of ATI cells ^2^. Our findings support these data, since the B2AE cell line in our co-culture loses CD74 expression and takes on a squamous appearance, simultaneously failing to form a sufficiently integrated layer to allow culture at ALI. Taking these comparisons into account, we plan to focus our efforts on the BATII cell line for our own studies, which rely on a maintenance of the ATII phenotype by a sub-population of the cells when cultured as part of a co-culture. We do, however, propose that other researchers may find the B2AE cell line useful, such as in studies where a native karyotype, a wild-type comparable proliferation rate and a single layer of cells is crucial.

The ability of the BATII cell line to maintain proliferative capacity and the expression of ATII markers, such as CD74 and SLC34A2, in 3D structures such as those observed in our Matrigel cultures alludes to their potential in spheroid and organoid studies of the alveolar epithelium, perhaps even more so than in co-culture. These spheroids were reminiscent of those reported by Lee *et al.* ^59^. As such, they may be of interest to researchers in the field of developmental biology, for example in exploring the mechanisms of alveolar differentiation via the Wnt pathway, building on previous organoid culture approaches ^60^. Previous literature reports the generation of organoids predominantly from sacrificed animals, as reviewed by Barkauskas *et al.* ^61^. Disadvantages of this approach include the cost and logistics of animal housing (a particularly significant hurdle when studying larger animals such as bovines), the ethical considerations of such methods due to the invasive nature of procedures used to simulate disease status and the reported necessity for mesenchymal ‘support’ cells for the formation of ATII-derived organoids in 3D culture ^62^. The BATII cell line derived spheroids require further characterisation, but we suggest that they offer much potential in the discipline of 3D organoid studies of the distal lung. Since our ongoing studies require the culture of cells at ALI as part of a co-culture, we have ceased to use Matrigel in our co-culture cultures, since the formation of organoids in this system causes the epithelial layer to peel back from the edges of the insert, causing ‘leakiness’ of the model.

The formation of an integral co-culture with BPAECs lends the BATII (and to an extent, the B2AE) cell line to pharmacological studies ^63^, or migration studies, including, but not exclusive to, neutrophils ^64^. No such studies have been performed in a bovine co-culture thus far, therefore we anticipate that the co-culture outlined here will facilitate bovine studies that parallel studies published previously for human *in vitro* models. It is important however, to weigh up the merits against the disadvantages of such an approach. The most obvious benefit to using cell lines, such as those in the current study, are the generation of identical populations, which help provide consistent and reproducible data that given careful control of variables, may be compared between groups. Immortalized cells tend to be easier to culture than cells used in primary cultures - they grow more robustly and do not require repeated isolations from living donors. An increased proliferation rate enables a higher throughput and the extraction of larger amounts for downstream assays.

The major disadvantage to using immortalized cells is that these cells divide indefinitely due to loss of control of the cell cycle. The use of viral oncogenes in particular results in loss of contact inhibition, as observed by the use of SV40 in the BATII cell line, and sometimes unique karyotypes not found *in vivo*. This may be counteracted to some extent by the co-introduction of hTERT with the viral oncogene, as performed by Zabner *et al* ^65^., whereby human bronchial epithelial (HBE) cells co-transfected with HPVE6/E7 and hTERT were genetically stable at passage 18, but still able to form integral tight junctions. Certainly this was the case in the BATII cell line (transduced with SV40 and hTERT). Like Zabner’s HBE cell line, we found that genomic instability emerged in later passages. Importantly, this did not affect the ability of the cell line to form tight junctions, or to differentiate. Transformation did increase the likelihood of multi-layered structures forming ( Figure 5A and Figure 6C), although it’s important to note that these structures did occur, albeit to a lesser extent, in wild-type ATII co-cultures (Dataset 6, see *Extended data*). In our studies, the multi-layered structures in the BATII co-cultures were surrounded by squamous layer of epithelial cells ( Figure 6E), which had lost the ATII markers studied. The aim when assembling our model was to generate a bilayer. In this respect, we were unsuccessful with regards to the BATII cell line.

The proposed model enables users to obtain representative alveolar co-cultures that retain certain desirable physiological and structural features of the native bovine alveolus within three weeks, whilst overcoming the challenges of housing large animals or the absence of large animal facilities. Most pertinent to the ethos of the 3Rs (in particular Replace), our co-culture model may be used as an *in vitro* alternative to *in vivo* experiments studying bovine respiratory diseases, for which 95 live cattle were used in the UK alone in 2016 ^29^. These include, but are not exclusive to, bovine tuberculosis, bovine respiratory disease and bovine syncytial virus. Our method could also be used in parallel with live animal studies. For comparison with an *in vivo* scenario, one recent study in which cattle were infected with live *M. tuberculosis* was conducted using 12 calves reared from birth to six months of age in bio-containment level 3 laboratory facilities. Cattle were culled ten weeks post infection ^66^, resulting in an overall study period of almost nine months. Whilst the number of live animals used may not be considered to be high, the authors would emphasise that the protracted nature of such experiments is a considerable drawback. It may also be argued that cattle used in such studies are not granted three of the ‘five freedoms of animal welfare’. These are “freedom from pain, injury or disease”, “freedom to express normal behaviour” and “freedom from fear and distress” ^25^.

Of particular interest to our group is the application of the model to study host-pathogen interactions in the bovine alveolus, between the ATII cell and *M. bovis*. Live animal studies have thus far provided a wealth of information, particularly those involving the natural target of *M. bovis*, the cattle themselves, as reviewed by Gregson *et al.* ^63^. The close relationship between *M. bovis* and *M. tuberculosis* implies that an *in vitro* bovine co-culture model would be of use in elucidating data for both pathogens and therefore enable comparative studies in disease pathogenesis. This has certainly been demonstrated by the evaluation of human treatments and vaccines in the bovine live animal model ^22, 23^. In order to accelerate advances in therapies and control strategies, however, a more fundamental understanding of early disease pathogenesis is urgently required. Early infection events are difficult to study in live animal models, whilst conducting real time studies using cattle housed in containment level 3 facilities is a logistical challenge.

One alternative approach to live animal models to study host-pathogen interactions is to follow an *ex vivo* approach, such as in Maertzdorf’s study of *M. tuberculosis* invasion of human lung explants ^67^. In a similar study, Ganbat *et al*. recently explored such interactions by a detailed comparison of various mycobacterial strains ( *M. tuberculosis, M. abscessus, M. avium*) ^68^. Explant studies such as these add considerable weight to the collective role of pulmonary cells including alveolar macrophages, monocytes, neutrophils and ATII cells, using studies with a comprehensive architecture that more accurately reflects the *in vivo* scenario. They are limited, however, to short term studies (16 hour post-infection time points in the case of Ganbat *et al.*).

A co-culture model would resolve some of the challenges and limitations of live animal models, whilst enabling longer term studies of biomarkers of pathogenesis; however they come with their own caveats. It is difficult, for instance to recapitulate the complete architecture of the alveolar epithelium *in vitro* and findings are limited to interactions with two cell types in the current study. Moreover, early host-pathogen interactions are not limited to the epithelium; numerous *in vivo* and *ex vivo* studies such as those above have demonstrated that an equally important role is played by various components of the immune system, in particular alveolar macrophages, as recently studied in human co-culture models of the alveolus ^69– 72^. Such studies also highlight the potential of co-culture models for studying interaction of the host epithelium with nanoparticles and aerosol medicines, in addition to disease pathogenesis. It is therefore imperative to consider the role of immune cells as part of such co-culture studies. The nature of our tissue source was prohibitive in this respect - the slaughter facility operates as a commercial meat processing business with limited access to post-mortem material. Whilst we are able to access PBMCs through our collaborators, tissue incompatibility caused by the use of different animals for construction of a model including immune cells may be a limiting factor. We would therefore concede that this is a drawback of the co-culture model proposed here. We are, however, performing studies using PBMC-conditioned media to assess whether or not it is even necessary to apply PMBCs directly to the co-culture.

To summarise, we present here two novel ATII derived cell lines, BATII and B2AE, together with a simple *in vitro* co-culture model of the bovine alveolus, including a detailed protocol by which other users can establish the model in their own institution. We have discussed the suitability of the immortalised BATII cells as organoid cultures to enable the translation of human developmental biology and tissue regeneration studies to the bovine model, whilst comparing and contrasting both cell lines generated by the lentiviral transduction of ATII cells with the viral oncogene SV40/hTERT combination (BATII), versus the proto-oncogene Bmi1 */*hTERT combination (B2AE). We have summarised how the use of the BATII cell line, in conjunction with the endothelial cell line BPAEC within a functional co-culture, could act as a viable alternative to live animal studies, overcoming logistical challenges associated with large animal models of disease pathogenesis. This applies particularly to pathogens such as *M. bovis* and *M. tuberculosis*, which require containment level 3 facilities. We propose that the co-culture model will allow the study of early host-pathogen interactions between the alveolar epithelium and the invading (respiratory) agent, including in the context of additional components of the innate immune system, such as neutrophils and macrophages. Such studies are extremely difficult to conceive in the *in vivo* setting due to the transient nature of such events. All of the components of the model are available either commercially (BPAECs, see ‘Culture conditions of BPAECs’) or through collaboration with the authors (BATII and B2AE cell lines). As such, other researchers are encouraged to consider this model as an alternative to live animal models. In doing so, there is considerable potential to advance the fields of respiratory disease in cattle, align research of bovine and human zoonoses and closely related pathogens in terms of preventative strategies as well as novel treatments and disease interventions. Consequently, the co-culture model offers great potential in replacing live animals used in the research of respiratory diseases affecting the distal airways. The authors are willing to discuss collaborations with other researchers wishing to use the bovine alveolar type II cell lines.

## Data availability

### Underlying data

Open Science Framework: f1000 research. https://doi.org/10.17605/OSF.IO/CXMBU ^42^

The project contains the following underlying data:

**-**Dataset 3_dextran blue assay.csv (Raw data underlying dextran blue permeability assay presented in Figure 7)
**-**Dataset 4_TEER.csv (Raw TEER values underlying Figure 7)
**-**Dataset 5_TEER_180619.jpg (Plotted TEER of mono-cultures compared with co-cultures, underlying discussion of TEER as a measure of integrity and use of different pore sizes)
**-** Dataset 7_TEER_individuals.csv (Raw TEER values underlying discussion of TEER as a measure of integrity)

### Extended data

Open Science Framework: f1000 research. https://doi.org/10.17605/OSF.IO/CXMBU ^42^

The project contains the following extended data:

**-**Dataset 1_3D BATII.mp4 (3D construction of the morphology of a BATII cell colony)
**-**Dataset 2_Z stack co-culture.mp4 (Z stack of a co-culture composed of an apical BATII epithelial layer overlaid onto BPAECs)
**-**Dataset 6_ATII_IF_180619.jpg (Z stack slices of ATII in co-culture with underlying endothelial BPAEC)

Data are available under the terms of the Creative Commons Zero "No rights reserved" data waiver (CC0 1.0 Public domain dedication).
