# Supplementary material for: A co-culture model of the bovine alveolus
Source: F1000Res. 2019 Jul 30;8:357. Originally published 2019 Apr 1. [Version 2] doi: 10.12688/f1000research.18696.2 (PMC6685456; doi:10.12688/f1000research.18696.2)
Supplement: Peer review history

# Peer review: Reviewer response for version 2

**DOI:** 10.5256/f1000research.21979.r51886

**Published:** 2019-08-12

**Authors:** Claus-Michael Lehr, Patrick Carius

**Affiliations:** 1 Helmholtz Institute for Pharmaceutical Research Saarland (HIPS), Helmholtz Centre for Infection Research (HZI), Saarland University, Saarbrücken, Germany; 2 Biopharmaceutics and Pharmaceutical Technology, Department of Pharmacy, Saarland University, Saarbrücken, Germany; 3 Helmholtz Institute for Pharmaceutical Research Saarland (HIPS), Helmholtz Centre for Infection Research (HZI), Saarland University, Saarland, Germany

The modified version of the manuscript is now approved without reservations.

Is the rationale for developing the new method (or application) clearly explained?

Yes

Is the description of the method technically sound?

Yes

Are the conclusions about the method and its performance adequately supported by the findings presented in the article?

Partly

If any results are presented, are all the source data underlying the results available to ensure full reproducibility?

Yes

Are sufficient details provided to allow replication of the method development and its use by others?

Partly

Reviewer Expertise:

Advanced Drug Delivery Technologies; cell and tissue models of epithelial barriers (intestines, skin, lung)

We confirm that we have read this submission and believe that we have an appropriate level of expertise to confirm that it is of an acceptable scientific standard.

# Peer review: Reviewer response for version 2

**DOI:** 10.5256/f1000research.21979.r51883

**Published:** 2019-08-12

**Authors:** Pietro Mastroeni

**Affiliations:** 1 Department of Veterinary Medicine, University of Cambridge, Cambridge, UK

Is the rationale for developing the new method (or application) clearly explained?

Yes

Is the description of the method technically sound?

Yes

Are the conclusions about the method and its performance adequately supported by the findings presented in the article?

Yes

If any results are presented, are all the source data underlying the results available to ensure full reproducibility?

Yes

Are sufficient details provided to allow replication of the method development and its use by others?

Yes

Reviewer Expertise:

Referee suggested by the NC3Rs for their scientific expertise and experience in assessing 3Rs impact

I confirm that I have read this submission and believe that I have an appropriate level of expertise to confirm that it is of an acceptable scientific standard.

# Peer review: Reviewer response for version 2

**DOI:** 10.5256/f1000research.21979.r51884

**Published:** 2019-08-06

**Authors:** Robert L Davies

**Affiliations:** 1 Institute of Infection, Immunity and Inflammation, College of Medical, Veterinary and Life Sciences, University of Glasgow, Glasgow, UK

The authors have addressed my suggestions in the initial review. I have no further comments.

Is the rationale for developing the new method (or application) clearly explained?

Yes

Are the 3Rs implications of the work described accurately?

Yes

Is the description of the method technically sound?

Yes

If applicable, is the statistical analysis and its interpretation appropriate?

Yes

Are the conclusions about the method and its performance adequately supported by the findings presented in the article?

Yes

If any results are presented, are all the source data underlying the results available to ensure full reproducibility?

Partly

Are a suitable application and appropriate end-users identified?

Yes

Are sufficient details provided to allow replication of the method development and its use by others?

Yes

Reviewer Expertise:

NA

I confirm that I have read this submission and believe that I have an appropriate level of expertise to confirm that it is of an acceptable scientific standard.

# Peer review: Reviewer response for version 1

**DOI:** 10.5256/f1000research.20472.r46561

**Published:** 2019-05-31

**Authors:** Robert L Davies

**Affiliations:** 1 Institute of Infection, Immunity and Inflammation, College of Medical, Veterinary and Life Sciences, University of Glasgow, Glasgow, UK

This paper describes the establishment of a co-culture bilayer model of the bovine alveolus using bovine pulmonary arterial endothelial cells (BPAECs) and immortalised bovine alveolar type II epithelial cells cultured for 14 days at an air-liquid interface (ALI). The authors highlight the practical, scientific and 3Rs benefits of their model and provide examples of current and potential applications.

 The rationale for establishing a bilayer alveolus model is well justified and the methodology is generally detailed and clear. The authors have chosen to create their bilayer by seeding the alveolar epithelial cells directly on top of confluent BPAECs and culturing at ALI (it is not clear whether an initial submerged phase was used which is normal practice). However, in previously established bilayer models of the alveolus (Hope *et al*., 2007 ^1^; Morton *et al*., 2014 ^2^; Costa *et al*., 2019 ^3^), the endothelial and epithelial cells have been grown on the basal and apical surfaces of the membrane, respectively. This better mimics the uninterrupted interaction between the endothelial cells and tissue fluids (i.e. blood). It is not clear why the authors have taken the approach they have, but I think this is an important point that should be highlighted with reference to other published studies and discussed. Was there a specific reason this approach was chosen? In relation to this, in the 5-day and 14-day cultures shown in Figure 1, cells are shown on the basal surface of the membrane; this is confusing. If these represent BPAECs that have migrated to the underside of the membrane, this needs to be explained because, in effect, a double endothelial layer has been created which is not representative of the *in vivo* situation. Is this related to the use of 8 mm pores (which seems very large) and could this have been avoided by the use of a smaller pore size (which were used in other experiments - see also comment below)?

 The reasoning behind the development of immortalized cell lines is logical although there are also disadvantages to this approach as demonstrated and discussed in the paper (the B2AE cell line, in particular, was not very successful). The imaging shown in Figure 3 highlights significant differences between the wild-type and immortalized cell lines grown on “coverglass”. 

 Unfortunately, wild-type primary alveolar epithelial cells do not appear to have been used in subsequent bilayer experiments which would have allowed important direct comparisons to have been made between these and the immortalized cell lines. In particular, the histological section of  the BATII/BPAEC layer shown in Figure 6C shows a thickened structure (and is certainly not pseudostratified) that is not representative of the native alveolar epithelium. The latter is obviously a very thin monolayer to allow for gaseous diffusion and, on this point, it would be helpful to have a figure of a histological section of *ex vivo* lung tissue showing the alveolar epithelium. Indeed, a more realistic single-cell layer is seen in the alveolar model created by Costa *et al*. (2019) ^3^. It would be helpful to readers and potential users of the cell lines for these points to be discussed.  In relation to these observations, the authors have switched between using membranes of different pore-diameters (0.4, 3.0 and 8 mm). Pore diameter is extremely important and selection is based on the experimental objectives. It is not clear why different pore size have been used and this could also be discussed.

Is the rationale for developing the new method (or application) clearly explained?

Yes

Are the 3Rs implications of the work described accurately?

Yes

Is the description of the method technically sound?

Yes

If applicable, is the statistical analysis and its interpretation appropriate?

Yes

Are the conclusions about the method and its performance adequately supported by the findings presented in the article?

Yes

If any results are presented, are all the source data underlying the results available to ensure full reproducibility?

Partly

Are a suitable application and appropriate end-users identified?

Yes

Are sufficient details provided to allow replication of the method development and its use by others?

Yes

Reviewer Expertise:

NA

I confirm that I have read this submission and believe that I have an appropriate level of expertise to confirm that it is of an acceptable scientific standard, however I have significant reservations, as outlined above.

## References

[1] : Pathogenesis of Aspergillus fumigatus and the kinetics of galactomannan in an in vitro model of early invasive pulmonary aspergillosis: implications for antifungal therapy. *J Infect Dis*.2007;195(3) : 10.1086/510535 455-66 10.1086/510535 17205486

[2] : Gene expression profiles of human dendritic cells interacting with Aspergillus fumigatus in a bilayer model of the alveolar epithelium/endothelium interface. *PLoS One*.2014;9(5) : 10.1371/journal.pone.0098279 e98279 10.1371/journal.pone.0098279 24870357PMC4037227

[3] : Triple co-culture of human alveolar epithelium, endothelium and macrophages for studying the interaction of nanocarriers with the air-blood barrier. *Acta Biomater*.2019;91: 10.1016/j.actbio.2019.04.037 235-247 10.1016/j.actbio.2019.04.037 31004840

# Response

**Published:** 2019-07-18

**Authors:** Diane Lee

**Affiliations:** University of Surrey, UK

Initial experiments did involve seeding cells on opposing sides of the membrane. Owing to difficulties generating quality sections of paraffin embedded membranes, we experimented with seeding both cell types on the apical side, as described by Birkness *et al*.(1999) and following personal communication with the corresponding author of that paper. Following this method allowed us to compare data, since our intention was to study the interaction of bovine TB with the alveolus, making our model the bovine equivalent of that of Birkness *et al*. On the point of mimicking the uninterrupted interaction between epithelial cells and endothelial cells, the authors suggest that in no *in vivo* situation will you find a synthetic membrane and that less interruption occurs when the cells are adjacent, rather than reaching through a pore. The large pores were necessary due to the intended use of the model, which would require mycobacteria and immune cells to migrate from one chamber to another. These points have now been discussed in the manuscript.

 The authors whole-heartedly agree that there are disadvantages as well as advantages to the use of immortalised cell lines. These have been included at length in the modified discussion.

 In light of the reviewer’s suggestions, we have included extended data with regards to the wild-type ATII as part of co-culture with endothelial cells (Dataset 6 shows IF images of z-stack slices, whilst dataset 7 includes raw data of the TEER values generated by culture and co-culture of each cell line and the wild-type ATII). The work of Costa *et al*. was published after the submission of version 1 of this manuscript, but has now been included in the discussion accordingly, as we agree that this is important to consider. We have also modified the manuscript discussion text to include justification for the different pore sizes. In brief, 0.4 was used to generate quality sections – a particular technical challenge, whilst 3 and 8 were used due to intended studies which included migration of PBMCs and mycobacteria (we notice that Costa *et al*. also use 3.0 μm, presumably for the same reason).

# Peer review: Reviewer response for version 1

**DOI:** 10.5256/f1000research.20472.r46560

**Published:** 2019-05-07

**Authors:** Claus-Michael Lehr, Patrick Carius

**Affiliations:** 1 Helmholtz Institute for Pharmaceutical Research Saarland (HIPS), Helmholtz Centre for Infection Research (HZI), Saarland University, Saarbrücken, Germany; 2 Biopharmaceutics and Pharmaceutical Technology, Department of Pharmacy, Saarland University, Saarbrücken, Germany; 3 Helmholtz Institute for Pharmaceutical Research Saarland (HIPS), Helmholtz Centre for Infection Research (HZI), Saarland University, Saarland, Germany

The authors provide a detailed guide to reproduce an *in vitro* tissue model of the bovine air-blood barrier, that consists out of bovine pulmonary arterial endothelial cells in co-culture with one out of two different immortalized bovine alveolar epithelial cell lines.

 The introduction puts the experiments in a concise but at the same well explained scientific context. The methods section is easy to follow and should allow for the reproduction of the presented model, although it could profit from some revisions:

- The diameter (mm) of all used Transwell growth supports should be exchanged by the growth area (cm²) to easily identify the growth area. 
- Measurement of cell layer integrity: It is unclear if pre-warmed medium was used to restore submerged conditions and then TEER was measured after 15 min of equilibration. In our case submerged conditions are restored by adding 200 µl apical and 600 µl basolateral (in case of 0.33 cm² inserts) of pre-warmed (37°C) medium to the ALI cultures. TEER values are then measured after at least 1 h of equilibration. The TEER measurement is performed on a heating plate (37°C), to prevent a fast temperature drop. 

Although a detailed overview of possible applications for this model to aid the 3R’s in future research is given be authors, the conclusions drawn from the displayed often appear not to be justified based on the data presented. This especially accounts for the results as well as the discussion section. The scientific benefits claimed in the “Research Highlights” appear to be exaggerated and are not based on the actually provided data.

 Results section:

- In accordance with the comments of Pietro Mastroeni, a scientific discussion of the selected markers is missing. Inspiration for discussion could be taken from references 1 ^1^ and 2 ^2^.
- Figure 6 A and B clearly show different magnifications
- Figure 6 C and D show the formation of multilayered epithelia and are not further discussed.
- BPAEC in Figure 7 A looks as the same as in Figure 7 B. Is it the same? Maybe combine into one figure.

Discussion section:

- The model is termed a “bilayer” of epithelial and endothelial cells. As shown in Figure 6 C and D, the BAT II culture shows the formation of multiple stacked layers, the B2AE culture at least more than two layers. This issue is not properly mentioned in the discussion.
- The development of TEER values greater than 1000 Ohm*cm² for the BATII cells, could be explained by the increased resistance of paracellular flux  based on the formation of functional tight junctions within a monolayer (as expected in vivo) or by an increase in resistance through the formation of multilayers. The formation of functional tight junctional complexes was in this study only indicated via TEER measurement and reduction of dextran permeability; immunohistochemistry or transcriptomic data are missing. Given the context of the massive multilayer formation shown in Fig. 6 C for the BATII culture, the missing information on tight junctional integrity would at least need a thorough discussion.
- The authors appear to be completely unaware of some quite relevant scientific work by other groups in the context of primary rat or human alveolar epithelial cells, resp. (See references 3 ^3^ and 4 ^4^) In the context of human alveolar epithelial cell lines the work of Tetley et al. ^5^ Kuehn et al. ^6^ should be considered. The same is true for the work as regards co-cultures of alveolar epithelial cells and immune cells by Rothen-Rutishauser et al. ^7^ Hittinger et al. ^8^ Kletting et. al. ^9^. A very recent paper by Costa et al. ^10^ describes a co-culture of human alveolar epithelial cells, endothelial cells and macrophages. 
- The whole discussion could benefit of an even more critical reflection of the advantages as well as disadvantages of the proposed model.

**Are a suitable application and appropriate end-users identified?**

*Yes*

**If applicable, is the statistical analysis and its interpretation appropriate?**

 Yes

**Are the 3Rs implications of the work described accurately?**

 Yes

Is the rationale for developing the new method (or application) clearly explained?

Yes

Is the description of the method technically sound?

Yes

Are the conclusions about the method and its performance adequately supported by the findings presented in the article?

Partly

If any results are presented, are all the source data underlying the results available to ensure full reproducibility?

Yes

Are sufficient details provided to allow replication of the method development and its use by others?

Partly

Reviewer Expertise:

Advanced Drug Delivery Technologies; cell and tissue models of epithelial barriers (intestines, skin, lung)

We confirm that we have read this submission and believe that we have an appropriate level of expertise to confirm that it is of an acceptable scientific standard, however we have significant reservations, as outlined above.

## References

[1] : Cross-Species Transcriptome Profiling Identifies New Alveolar Epithelial Type I Cell-Specific Genes. *Am J Respir Cell Mol Biol*.56(3) : 10.1165/rcmb.2016-0071OC 310-321 10.1165/rcmb.2016-0071OC 27749084PMC5359537

[2] : Long-Term Culture of Distal Airway Epithelial Cells Allows Differentiation Towards Alveolar Epithelial Cells Suited for Influenza Virus Studies. *EBioMedicine*.2018;33: 10.1016/j.ebiom.2018.05.032 230-241 10.1016/j.ebiom.2018.05.032 29937069PMC6085545

[3] : Isolation and culture of alveolar type II cells. *Am J Physiol*.1990;258(4 Pt 1) : 10.1152/ajplung.1990.258.4.L134 L134-47 10.1152/ajplung.1990.258.4.L134 2185652

[4] : *Pharmaceutical Research*.1999;16(5) : 10.1023/A:1018887501927 601-608 10.1023/A:1018887501927

[5] : Immortalization of human alveolar epithelial cells to investigate nanoparticle uptake. *Am J Respir Cell Mol Biol*.2008;39(5) : 10.1165/rcmb.2007-0334OC 591-7 10.1165/rcmb.2007-0334OC 18539954PMC2643209

[6] : Human alveolar epithelial cells expressing tight junctions to model the air-blood barrier. *ALTEX*.2016;33(3) : 10.14573/altex.1511131 251-60 10.14573/altex.1511131 26985677

[7] : A three-dimensional cellular model of the human respiratory tract to study the interaction with particles. *Am J Respir Cell Mol Biol*.2005;32(4) : 10.1165/rcmb.2004-0187OC 281-9 10.1165/rcmb.2004-0187OC 15640437

[8] : Autologous co-culture of primary human alveolar macrophages and epithelial cells for investigating aerosol medicines. Part I: model characterisation. *Altern Lab Anim*.2016;44(4) :337-347 2768518510.1177/026119291604400404

[9] : Co-culture of human alveolar epithelial (hAELVi) and macrophage (THP-1) cell lines. *ALTEX*.2018;35(2) : 10.14573/altex.1607191 211-222 10.14573/altex.1607191 29169185

[10] : Triple co-culture of human alveolar epithelium, endothelium and macrophages for studying the interaction of nanocarriers with the air-blood barrier. *Acta Biomater*.2019; 10.1016/j.actbio.2019.04.037 10.1016/j.actbio.2019.04.037 31004840

# Response

**Published:** 2019-07-18

**Authors:** Diane Lee

**Affiliations:** University of Surrey, UK

The authors have modified the manuscript to include the surface area of the Transwell inserts, in addition to the diameter throughout the manuscript. In performing TEER measurements, medium was pre-warmed (in the incubator). We do not have a heating plate and therefore some loss of temperature was inevitable. To help counteract this, we take three measurements from all wells (for each measurement working from the first to last sample before starting again at the first), to monitor the extent of TEER change with temperature. This has been clarified in the methods. We did calculate drift for eight inserts between the first and last measurements. In these calculations, TEER increased between 3 and 25 % (Mean 9 ± 8 %) for the BATII co-cultures and 3 and 22 % (Mean of 7 ± 6 %) for B2AE co-cultures respectively. Drift was highest in the earlier measurements (day 3) in both cases.

 Until co-culture studies using the proposed model are performed at length and data from multiple groups compared, it’s not possible to say for sure how much of a benefit any model will provide to the field. The authors continue to refine the model and encourage others to collaborate or make use of this approach in their own studies. With regards to scientific benefits, there are studies which simply cannot be performed in live bovines at present; these particularly include the early (and transient) events which occur during infection with pathogens such as M.bovis, or RSV. We hope that the benefits of such an approach as the one proposed in the current paper are clarified by our extended discussion.

 We have further clarified why we chose the markers used in this study and included further discussion as appropriate. We thank the observant reviewer for highlighting the discrepancy in Figure 6 and have corrected the figure accordingly. With regards to the comments on Figure 6 C and D, we have added to both the results section and discussion section with regards to the multi-layered epithelial layers in the bilayers generated by BATII. These were isolated in the ALI cultures, their presence being easily identified using x-z confocal microscopy, as shown in Figure 5 and the extended data. We have discussed these morphological anomalies further in the text. The data for BPAEC in Figure 7A and 7B are from different experiments/sets of data. The actual values are 55.7 ± 5.2 and 48 ± 3.4 % respectively.

 The authors concede that in areas, the model is more than two cells thick, although this is not universal across the membrane (shown by the x-z confocal images of Figure 5 and extended data). We have included a further image (E) in Figure 6 to put these 3D structures in context and expanded the discussion accordingly, including whether it would be more accurate to describe the model as ‘co-culture’. It should be noted, however, that in our studies, the wild type ATII cells also formed 3D structures when grown on membranes in co-culture with BPAEC (see Dataset 6, which shows frames from an ATII/BPAEC co-culture Z-stack). This occurred whether in co-culture with the endothelial BPAEC, as in the current study, or when seeded as a mono-culture, as in our previous paper (see Lee et al. 2018). We accept that the use of the term ‘bilayer’ in this instance is not accurate and have modified the manuscript to describe the model as a co-culture, rather than a bilayer.

 TEER values of >1000 were also generated for wild-type ATII co-cultures with endothelial cells, which we have included in the extended data (Dataset 5 and 7). As clarified above (and further clarified in the text), the 3D structures were not uniform in thickness. The thickness ranged from one cell upwards. It is our experience that TEER values represent the area of the lowest integrity in the culture i.e. the thinnest/with the fewest number of functional tight junctions. This is evidenced by the drop in TEER in cultures damaged by experimental manipulations (scrape by pipette and so on). Dataset 5 contains raw includes raw TEER data for BATII/B2AE only, BPAEC only, WT only and each as part of a co-culture of epithelial/endothelial cells, plotted as dataset 7. We have not included IHC data as the antibodies we tried (ZO-1 and Occludens) were cross-reactive with other antigens or generated high background signal. This lowered our confidence in their suitability for their use in the characterisation of the model.

 The authors are indeed aware of the work of Kemp *et al*. (referred to as Tetley above). Whilst they do consider the work relevant to discussions of immortalised cell lines, Kemp *et al*. transduced a temperature sensitive mutant of SV40 and cultured the resulting cells without other cell types. We have extended the discussion to further consider this and the work of other groups. We have recently completed studies of the interaction of the co-culture model of the current study with immune cells (PBMCs). As the current paper is a methods paper, we focussed here on the setup of the model and a manuscript detailing our latter work is in preparation.

# Peer review: Reviewer response for version 1

**DOI:** 10.5256/f1000research.20472.r46557

**Published:** 2019-04-05

**Authors:** Pietro Mastroeni

**Affiliations:** 1 Department of Veterinary Medicine, University of Cambridge, Cambridge, UK

The paper describes the generation and characterisation of two immortalised bovine epithelial cell lines to be used in conjunction with bovine arterial endothelial cells for the creation of structures that would model the alveolar environment of bovine species. The authors very clearly list the potential applications of such approach and its impact on the 3Rs. It would be desirable to explain in more depth what is the incontrovertible evidence that such models are truly representative of an *in vivo* situation.

 The introduction is very clear and comprehensive and allows the reader to gain information on the background to the study and on the general context of the experiments.

 The methods section is also clear and detailed, thus allowing reproducibility of the experiments.

 There are a few points that the authors might want to address in their revision of the manuscript.

1. Figure 3. The pictures relative to the wild type cells are hugely different from the ones obtained when using either of the cell lines. This might be confusing for the reader who may wonder how such large morphological differences can then lead to the conclusion that the immortalised cells lines are a good model for the natural alveolar environment. Perhaps this needs to be explained more clearly and discussed in detail.
2. In some sections the authors describe results for only one cell line (e.g. Matrigel cultures) whilst in others they cover both cell lines. This may be confusing.
3. A clear explanation of the choice of the cell markers is not always provided in the results section. This would make the manuscript clearer. 
4. It might be helpful to have a conclusive statement at the end of each section of the results to state the main findings and conclusions of that particular set of experiments. Similarly, a sentence introducing each section and explaining why the experiments were done would add clarity. This is done for some results sections but not for others. Therefore, sometimes the reader is left to wonder....why are they doing this?
5. It might be useful to show some histological and immune-fluorescence pictures of alveolar structures from a bovine lung to show the extent of similarities and differences between the proposed approach and the mammalian alveolar structure.
6. Alveolar macrophages (together with other immune cells) are a key to lung homeostasis and protection against infectious diseases. It needs explaining how structures devoid of such important immune cells can be considered representative of an *in vivo* situation, especially when one of the intended uses would be the study of infectious diseases.
7. The approach described in this study entails the generation of cell lines from a single animal. How can the authors be sure that these cell lines would be representative especially when dealing with a species that is clearly outbred. What are the dangers of animal-to-animal variability and therefore lack of representativeness?

**Are a suitable application and appropriate end-users identified?**

*Yes*

**If applicable, is the statistical analysis and its interpretation appropriate?**

 Yes

**Are the 3Rs implications of the work described accurately?**

 Yes

Is the rationale for developing the new method (or application) clearly explained?

Yes

Is the description of the method technically sound?

Yes

Are the conclusions about the method and its performance adequately supported by the findings presented in the article?

Yes

If any results are presented, are all the source data underlying the results available to ensure full reproducibility?

Yes

Are sufficient details provided to allow replication of the method development and its use by others?

Yes

Reviewer Expertise:

Infection, Immunity, Histopathology, tissue microbiology, molecular biology, vaccines, antibiotics. Referee suggested by the NC3Rs for their scientific expertise and experience in assessing 3Rs impact

I confirm that I have read this submission and believe that I have an appropriate level of expertise to confirm that it is of an acceptable scientific standard, however I have significant reservations, as outlined above.

# Response

**Published:** 2019-07-18

**Authors:** Diane Lee

**Affiliations:** University of Surrey, UK

The authors acknowledge that here, as in many cell lines, there are morphological differences when compared to the wild type when grown on coverglass. We attributed this to the increased proliferation rate of the BATII cell line and the lack of contact inhibition, characteristic of SV40 Large T Antigen transformed cells. The B2AE cell line was slower by comparison and did not form multi-layered structures on coverglass. Although we still observed multilayer structures in co-cultures generated using the BATII cell line (and to a lesser extent, the wild-type ATII), these covered <20 % of the culture surface, being surrounded by thinner mono/bilayers of epithelial-like cells. We have expanded the results section to clarify and also included a discussion of the advantages versus disadvantages of using cell lines which differ in phenotype from their wild-type counterparts.

 The omission of the B2AE cell line from some experiments was due to the growth rate of B2AE being very slow and not generating enough stocks to perform experiments with meaningful numbers of replicates. We chose to therefore focus on the BATII cell line for some experiments and have clarified as such in the text.

 We have added explanations or further clarification to justify choice of marker in each instance, along with references where necessary. Conclusive statements have also now been included or extended for the results sections, along with the inclusions of an introductory sentence or two.

 We have included an H&E image and highlighted differences/similarities between the proposed model and alveolar architecture in discussion. In addition, the inclusion of immune cells has been added to the discussion – studies including peripheral blood mononuclear cells (PBMCs) have been performed and are being published separately as part of further studies of M.bovis-BCG interaction with the bovine alveolus (manuscript in preparation).

 Lastly, the authors concede that this is from a single animal and therefore cannot be entirely representative of a whole species or even breed. We have added further to the discussion to this effect, with additional emphasis on the positives and negatives of the utilisation of cell lines when modelling the in vivo situation. We have also published detailed protocols of the isolation procedure, enabling researchers to isolate cells from multiple animals and compare phenotypes.
